# eHealth Approaches to Multimorbidity Management: Scoping Review of Interventions and Multidisease Strategies

**DOI:** 10.2196/88021

**Published:** 2026-09-08

**Authors:** Katja Bochtler, Salima Houta

**Affiliations:** 1 Faculty of Computer Science, Kempten University of Applied Sciences, Bahnhofstr. 61, Kempten, Bavaria, 87435, Germany, 49 8312523-641; 2 Bavarian Center for Digital Health and Social Care, Kempten, Bavaria, Germany; 3 Department of Computer Science, TU Dortmund University, Dortmund, North Rhine-Westphalia, Germany

**Keywords:** multimorbidity, digital health, eHealth intervention, integrated care, multidisease approaches, disease interactions, self-management

## Abstract

**Background:**

Multimorbidity, defined as the coexistence of two or more chronic conditions, challenges traditional, single-disease health care models. Although digital health technologies are increasingly applied to complex patient care, most interventions remain disease-specific and fail to account for interactions between coexisting conditions.

**Objective:**

This scoping review aimed to systematically map and synthesize existing evidence on digital health interventions that explicitly address multimorbidity, focusing on how these solutions integrate disease interdependencies between coexisting diseases in their design, implementation, and evaluation. We aimed to identify underlying concepts, map frequently targeted disease clusters, and assess reported outcomes and barriers to real-world application.

**Methods:**

Following the PRISMA-ScR (Preferred Reporting Items for Systematic Reviews and Meta-Analyses extension for Scoping Reviews) framework and informed by PRISMA-S (Preferred Reporting Items for Systematic Reviews and Meta-Analyses literature search extension) recommendations, we conducted systematic searches in PubMed and CINAHL for studies published in English or German between January 2014 and May 2024. Eligible studies described digital health interventions targeting patients with two or more conditions and explicitly addressed interactions among diseases, symptoms, or treatments. We strictly excluded studies of digital solutions that addressed multiple conditions through isolated modules or separate functionalities without cross-condition integration. In addition, research focusing solely on technical development, polypharmacy, or usability was excluded. Two reviewers independently screened records and extracted data using a structured charting form. Data were synthesized using descriptive statistics and thematic analysis.

**Results:**

Of 1660 records identified, 11 studies representing seven distinct digital health projects met the inclusion criteria. Most interventions focused on older adults with cardiometabolic or respiratory multimorbidity and combined mobile apps, telemonitoring platforms, and decision-support or analytic modules. While most interventions demonstrated feasibility and user acceptance, only a minority operationalized disease interactions beyond aggregated data. Approaches to multimorbidity included cross-disease analytics, composite risk indicators, and guideline-based decision support; however, systematic reconciliation of competing clinical recommendations was rare. Evaluation outcomes mainly addressed usability, engagement, and feasibility, whereas evidence regarding clinical effectiveness remained limited and inconsistent. Only two studies assessed clinical outcomes, with mixed findings.

**Conclusions:**

This review provides a novel synthesis of how digital health interventions explicitly address multimorbidity as an interconnected phenomenon rather than as a set of isolated conditions. Current evidence suggests feasibility and user acceptance of digital tools, whereas robust implementation of multimorbidity-aware functionalities and evidence regarding benefit remain limited. Advancing this field will require interoperable, data-integrative systems capable of supporting coordinated multimorbidity care across conditions and care settings. These findings highlight the need for a shift from disease-centric toward integrated multimorbidity-oriented digital care models.

## Introduction

### Background

Multimorbidity, commonly defined as the cooccurrence of two or more chronic conditions in an individual [1], represents one of the most significant and complex challenges facing contemporary public health systems. Its global prevalence has risen substantially due to population aging and lifestyle-related risk factors. Although multimorbidity is more frequent in older adults, more than 50% of individuals affected are under the age of 65 years [2]. A recent meta-analysis estimated the global prevalence of multimorbidity at 37.2%, with the highest rates in South America (45.7%), followed by North America (43.1%), Europe (39.2%), and Asia (35%) [3]. The impact of multimorbidity extends far beyond individual diagnoses, affecting physical function, quality of life, mental health, and mortality [2,4]. Managing multiple long-term conditions often involves complex and conflicting self-care regimens, frequent clinical appointments, and polypharmacy [5,6]. These interconnected health demands often surpass the capacity of current health care systems, which remain predominantly organized around single-disease paradigms [2,4,7,8]. There is increasing recognition of the need to transition from disease-centered to patient-centered care. Effective management of multimorbidity demands integrated, personalized, and coordinated care strategies that address the complex interplay of biological, psychological, and social dimensions of health [8,9].

Digital health technologies can support person-centered care. They offer scalable, flexible, and low-threshold access to care and information, particularly in resource-constrained environments. However, most existing solutions continue to focus on single diseases, often replicating the same disease-centric logic that dominates conventional health care [9,10]. While these applications may offer high usability and clinical value for specific conditions, they often overlook the multifaceted needs of patients with multimorbidities. Physicians have also expressed concern that many health apps and electronic health records remain fragmented and condition-specific, thereby impeding coordinated and holistic care [11,12], which directly contradicts European policy frameworks promoting integrated, cross-sectoral patient care and shared decision-making [13]. As a result, patients with multimorbidities often face the burden of navigating multiple disease-specific apps, platforms, and care plans.

### Rationale

Previous reviews examined specific comorbidity pairs and digital health interventions across diseases, including communication tools, self-management support, telemedicine, and clinical decision support systems (CDSSs). Yet, evidence for truly integrated digital support in multimorbidity remains limited, as most studies addressed conditions in parallel rather than their interactions [14-22].

None of the studies examined how interventions operationalize disease interdependencies, such as modeling condition interactions or reconciling competing treatment recommendations, which is an essential feature of truly integrated multimorbidity care. Given the fragmented and heterogeneous nature of the literature, a scoping review was considered the most appropriate methodology.

### Objectives

This scoping review synthesizes evidence on digital health solutions for multimorbidity management that explicitly address interactions between coexisting conditions. The primary and secondary objectives are given in Textbox 1.

Textbox 1. **Primary and secondary objectives.**

**Primary objectives**

- To identify and classify eHealth solutions for multimorbidity, focusing on cross-disease interactions and symptom interdependencies.
- To analyze underlying concepts, including strategies for integration, personalization, and care coordination.
- To map targeted diseases or multimorbidity clusters and identify recurring patterns.

**Secondary objectives**

- To assess reported effectiveness and implementation outcomes, including feasibility, usability, and clinical impact.
- To identify challenges and barriers to real-world scalability and sustainability.

## Methods

### Protocol and Registration

This scoping review was conducted in accordance with the PRISMA-ScR (Preferred Reporting Items for Systematic Reviews and Meta-Analyses extension for Scoping Reviews [23]; see Checklist 1 for the completed report and abstract checklists). The reporting of the search strategy was additionally informed by the PRISMA-S (Preferred Reporting Items for Systematic Reviews and Meta-Analyses literature search extension) to enhance transparency and reproducibility. A formal review protocol was not prospectively registered.

### Eligibility Criteria

Eligibility criteria were established a priori to ensure a systematic and targeted selection of relevant studies (Table 1). To ensure methodological rigor, the eligibility criteria were structured according to the Population, Concept, and Context (PCC) framework [24].

**Table 1.** Eligibility criteria using the Population, Concept, and Context framework.

Domain	Inclusion criteria	Exclusion criteria
Population	Individuals with clinically diagnosed multimorbidity (≥2 chronic conditions) Studies involving health care providers using multimorbidity cases (eg, vignettes and simulated profiles)	Single-disease populations Frailty-only populations without confirmed multimorbidity
Concept	Digital health interventions for treatment, management, or monitoring of multimorbidity Multicondition functionality addressing disease interactions	No digital or eHealth component (eg, purely face-to-face care, paper-based interventions, telephone-only interventions without digital integration) Single-disease interventions (even in multimorbid populations) Technology used only for data collection and analysis No explicit focus on disease interactions Articles focusing solely on usability, usage behavior, polypharmacy, or cost analysis Technical development studies without clinical, patient-centered, or system-level context
Context	No restriction on application settings or geographic and cultural background	No clear link to clinical multimorbidity management (eg, abstract models without application context for multimorbidity)
Types of evidence sources	Peer-reviewed primary studies	Editorials, commentaries, reviews, and protocols Conference abstracts without full text Unavailable full texts
Language	English German	Other languages
Date range	Studies published between January 1, 2014, and May 5, 2024	Studies published outside date range

### Information Sources

Systematic searches were conducted in PubMed and CINAHL for studies published between January 1, 2024, and May 5, 2024. These databases were selected because they provide comprehensive coverage of biomedical, clinical, and health services research, including nursing and care-related literature, which is particularly relevant for multimorbidity and integrated care contexts. Multidatabase platforms were not used for this search. Although the search was limited to two major databases, additional backward reference screening of included studies and relevant reviews was conducted to improve coverage. Searches were limited to English- and German-language publications. The final search was conducted on May 5, 2024.

Other methods, including the search of study registries, online resources, or handsearching of specific journals, were not part of the research methodology. No direct contact with authors was established.

### Search

The search strategy was developed iteratively to balance sensitivity and specificity, informed by established guidance for evidence synthesis. Search terms combined concepts related to digital health (eg, eHealth, mobile health [mHealth], and telemedicine) and multimorbidity (eg, comorbidity and multiple chronic conditions).

To enhance completeness, a multistep approach was applied:

1. Identification of key publications through preliminary searches and prior domain knowledge.
2. Analysis of keywords and indexing terms from relevant studies to identify additional synonyms and related concepts.
3. Use of a general-purpose large language model (LLM; ChatGPT, OpenAI) during the exploratory development of the search strategy to generate additional candidate synonyms for search terms. The LLM was used exclusively to support terminology expansion; all generated terms were manually reviewed by the authors and only those deemed relevant were incorporated into the final search string.
4. Iterative testing and refinement of the search query in both databases with manual screening of results to optimize relevance.

The final search strings for each database are provided in Multimedia Appendix 1. Searches were conducted in titles, abstracts, and keywords in PubMed and in CINAHL in all text fields.

No prevalidated search filters were applied. This search was an original effort and did not build upon prior work or previous search updates. A formal peer review of the search strategy was not conducted.

### Selection of Sources of Evidence

All references from the literature search were imported into a Citavi citation manager library (Lumivero). Deduplication was performed by comparing DOIs and titles. The screening process used the following steps:

1. Each reviewer independently screened all titles and abstracts against the defined eligibility criteria. A small subset of studies was reviewed jointly in advance to calibrate the reviewers and ensure a shared understanding and consistent application of the eligibility criteria, particularly regarding technologically supported disease interactions in multimorbidity management.
2. All articles deemed potentially relevant were retrieved in full and independently assessed by both reviewers.

Disagreements at both stages were resolved through discussion and consensus.

### Data Charting Process

Data was extracted using a structured spreadsheet developed for this review. Two reviewers (KB and SH) independently charted the data and discussed uncertainties throughout the process to ensure consistent interpretation of the charting items. The charting form was refined iteratively as familiarity with the evidence increased.

### Data Items

The extracted items were organized into five main domains: (1) general information (eg, authors, year, and country), (2) disease characteristics (eg, targeted diseases and degree of multimorbidity), (3) digital solutions (eg, type of technology and integration mechanisms), (4) evaluation methods (eg, study design and sample demographics), and (5) outcomes (eg, primary and secondary outcomes). A detailed list of all extracted data items is provided in Multimedia Appendix 2.

### Synthesis of Results

Charted data were summarized descriptively using frequencies, ranges, and medians where appropriate. In addition, intervention characteristics were analyzed thematically and grouped into recurring categories relating to disease interactions, technological functionalities, care concepts, implementation approaches, and reported outcomes. This facilitated the identification of common patterns and differences across digital interventions for multimorbidity.

## Results

### Selection of Sources of Evidence

**Figure 1.**
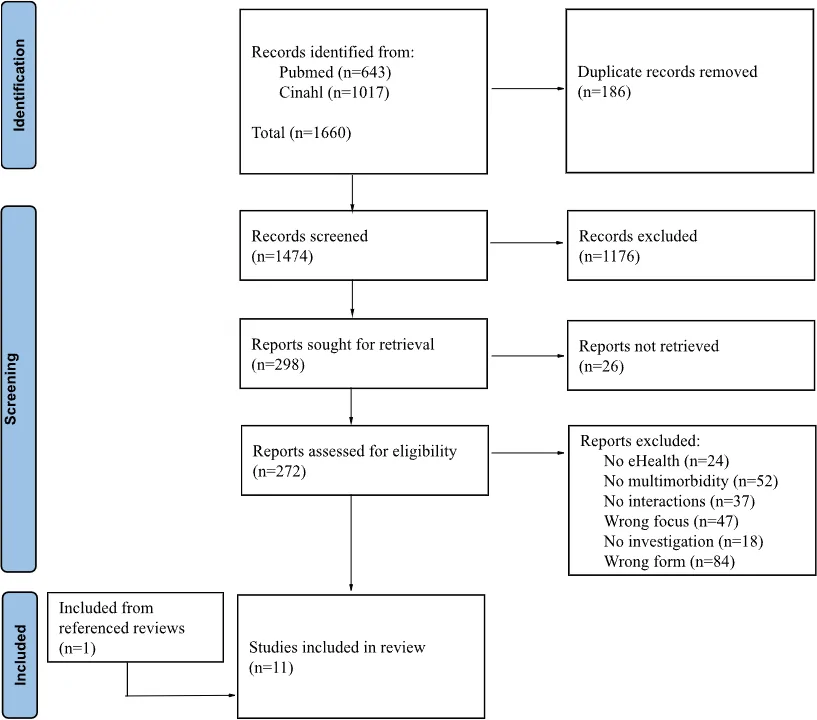
PRISMA-ScR (Preferred Reporting Items for Systematic Reviews and Meta-Analyses extension for Scoping Reviews) flow diagram of the study selection process for a scoping review of interventions and multidisease strategies accounting for disease interactions in multimorbidity (2014‐2024).

A total of 1660 studies were identified in our search across two databases: 643 (38.7%) from PubMed and 1017 (61.3%) from CINAHL. After removing duplicates, 1474 (88.8%) studies remained. Screening of abstracts and titles excluded 1176 (79.8%) studies that did not meet the inclusion criteria. Of the remaining 298 studies, 26 (1.57%) full-text papers could not be retrieved. Thus, 272 papers underwent a full-text review. Following this assessment, 10 (0.6%) studies met the inclusion criteria, and one relevant study was identified through screening of reference lists of review articles [25-35]. In total, 11 papers [25-35] were included in this review. The PRISMA diagram systemizing the screening process can be seen in Figure 1, as well as the reasons for full-paper exclusion of 262 (15.78%) studies.

### Characteristics of Sources of Evidence

In total, 11 studies [25-35] were included in this review. These are linked to seven unique digital health projects: Integrated Technology Systems for Proactive Patient-Centered Care (ProACT [25-27]), Mobile Airways Sentinel Network (MASK [28-30]), Multimorbidities Managing Technology for Healthcare (MATCH) [31], Autonomie trotz Multimorbidität in Sachsen durch Patientenempowerment, Holistische Versorgung für Ältere mit Vernetzung aller Regionalen Einrichtungen und Dienstleister (Autonomy despite Multimorbidity in Saxony through Patient Empowerment: Holistic Care for Older Adults via the Networking of All Regional Facilities and Service Providers; ATMoSPHÄRE [32]), Novel Model for Health Care Delivery (NOMHAD [33]), Mobile Atrial Fibrillation Application (mAFA [34]), and An integrated mHealth System for the Prevention and Care of Chronic Disease (mWellcare [35]).

**Figure 2.**
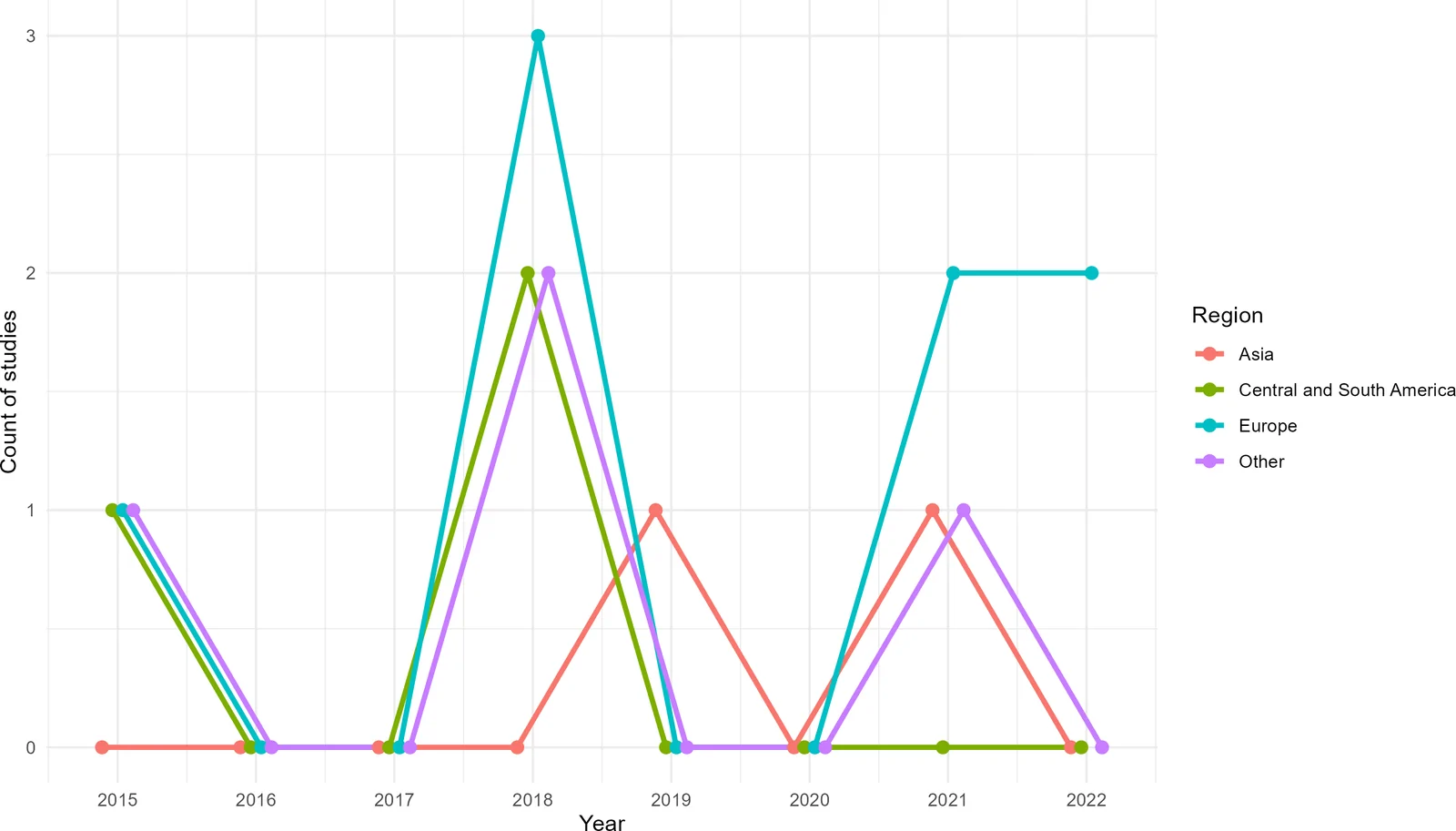
Annual number of publications on digital support accounting for disease interactions in multimorbidity, by region (N=11; 2014‐2024).

The majority of studies included in this scoping review were conducted in European countries. Specifically, out of the 11 included studies, 9 (81.82%) [25-33] studies involved European countries, with common contributors being Ireland, Belgium, Germany, and France. Other represented regions included Asia (eg, China and India), Central and South America (eg, Mexico and Brazil), and Oceania (Australia; Figure 2).

The number of included studies did not show a clear upward trend over time. While individual years such as 2018 and 2021 show a higher number of publications, the distribution across years remains relatively flat, suggesting that this research topic is not yet growing rapidly in the literature or is possibly underrepresented.

### Results of Individual Sources of Evidence

Data extraction was conducted on 2 levels: study-specific information such as study design, sample size, and outcomes were documented per publication. In cases where multiple publications referred to the same project, data extraction was partially aggregated. The description of addressed diseases, target groups, technologies, functionalities, and data categories were conducted on a project level to capture the overall context and structure of the eHealth intervention. Study-specific and project-specific data extractions can be found in Multimedia Appendices 3 and 4.

### Disease Characteristics

The seven projects included in the reviewed studies cover a range of chronic conditions. mAFA had the highest diversity of disease mentions (n=6), encompassing conditions such as atrial fibrillation (AF), coronary heart disease (CHD), congestive heart failure (CHF), depression, prior ischemic stroke, and pulmonary disease. Other projects such as ProACT (n=5) and mWellcare (n=4) also featured multiple diseases, indicating a broader scope in terms of comorbidity inclusion. In contrast, projects like MASK and MATCH featured the combination of two specific conditions. Although MASK also encompasses disease heterogeneity, it was considered eligible for this review because the intervention explicitly addresses the coexistence and interactions of asthma and allergic diseases within integrated multicondition care pathways rather than managing a single disease in isolation. The project ATMoSPHÄRE is not restricted to specific diseases or symptoms. The recommendations within this project are not medical in nature but rather focus on social services, including improving the overall condition and management of the disease, rather than recommending treatment.

CHF (n=5) and diabetes (n=4) were the most frequently addressed diseases, often appearing together in studies focused on cardiovascular or metabolic multimorbidity. Chronic obstructive pulmonary disease (COPD; n=3) was the third most frequently mentioned condition.

**Figure 3.**
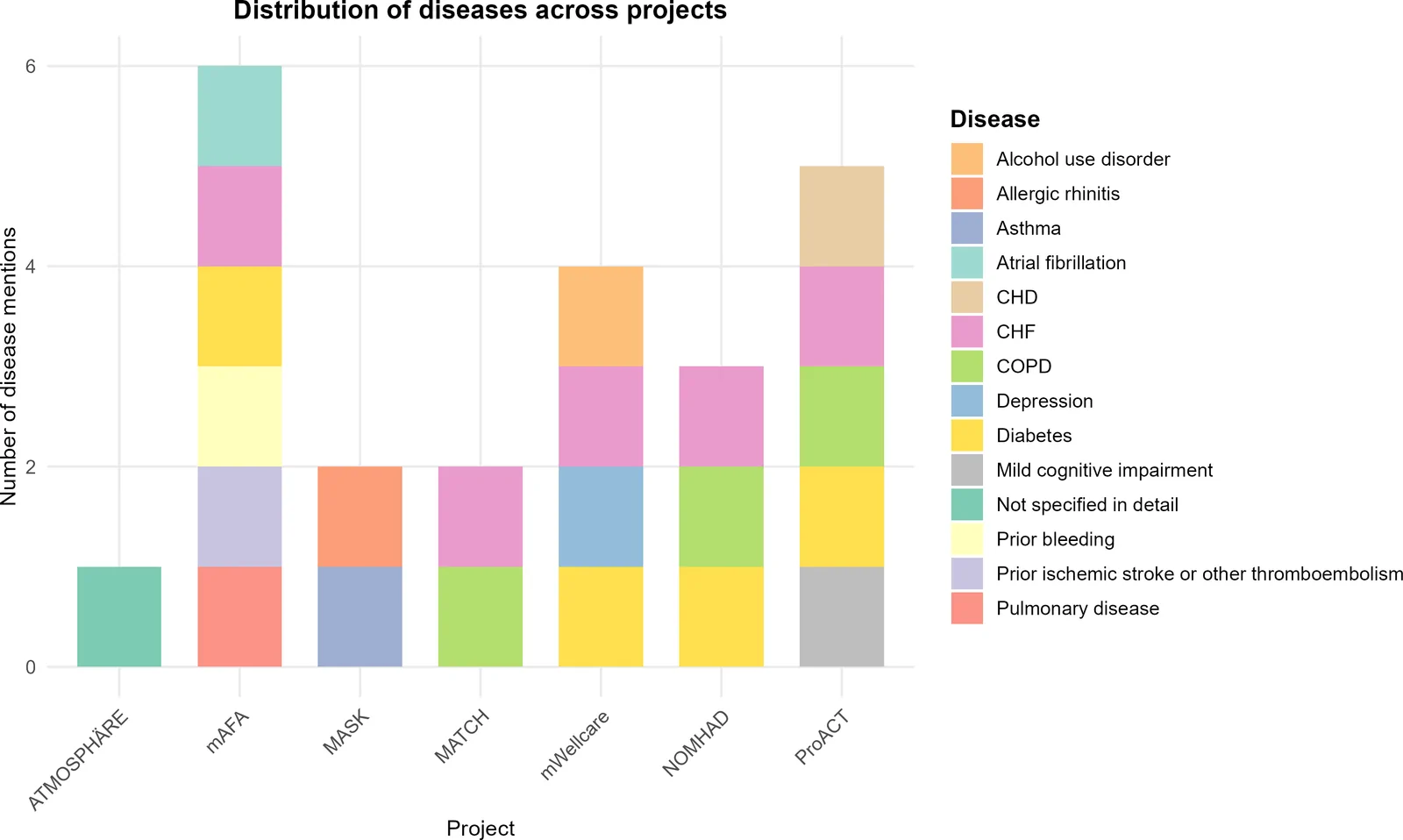
Diseases targeted by digital health projects (n=7) accounting for multimorbidity interactions, derived from the included studies (N=11; 2014‐2024) [25-35]. ATMoSPHÄRE: Autonomy despite multimorbidity in Saxony through patient empowerment: Holistic care for older adults via the networking of all regional facilities and service providers.; CHD: coronary heart disease; CHF: congestive heart failure; COPD: chronic obstructive pulmonary disease; mAFA: Mobile Atrial Fibrillation Application; MASK: Mobile Airways Sentinel Network; MATCH: Multimorbidities Managing Technology for Healthcare; mWellcare: an integrated mHealth system for the prevention and care of chronic disease; NOMHAD: Novel Model for Health Care Delivery; ProACT: Integrated Technology Systems for Proactive Patient-Centered Care.

The seven reviewed projects address varying levels of multimorbidity, reflecting different degrees of complexity in chronic disease management. Three projects (MASK, MATCH, and mWellcare) focused on specific combinations of two diseases, typically addressing well-defined comorbidity constellations such as cardiometabolic or respiratory disorders. These represent more straightforward cases of multimorbidity where clinical pathways and treatment strategies are relatively well established. In two projects (ProACT and NOMHAD), participation required the presence of two or more chronic conditions. For example, in ProACT, participants need to have at least two diseases out of a predefined disease set (CHD, CHF, COPD, diabetes, and mild cognitive impairment; Figure 3). This broader inclusion criterion reflects a more flexible design, capable of accommodating a wider range of patient needs while still maintaining a manageable level of system complexity. In one study (mAFA-Trial), inclusion required at least three chronic conditions from a predefined disease set, addressing a higher degree of multimorbidity and the corresponding need for more integrated care and decision support. Only one study (ATMoSPHÄRE) imposed no restrictions on the type of supported conditions, requiring only the presence of multimorbidity rather than specific disease combinations. Instead of providing disease-specific recommendations, it offered supportive interventions based on comprehensive geriatric assessments.

### Classification of eHealth Solutions

The classification of the eHealth interventions presented in the included studies was conducted from both a technical and functional perspective. For the categorization, we considered the World Health Organization (WHO) Classification of Digital Health Interventions v.1.0 as a foundational framework [36] but developed custom taxonomies to better capture the technological and functional aspects relevant to our analysis. In terms of technology type, we found five relevant system categories represented in the papers: (1) mHealth application for patient use, (2) home-based wearable and sensor devices, (3) health data infrastructure and telemedicine applications for health care providers, (4) health data processing and analytic modules, and (5) digital recommendation systems (DRSs).

In terms of which functions are supported by the technology, we found five categories: (1) self-management; (2) remote monitoring; (3) patient communication, engagement, and education; (4) integrated care and care coordination; and (5) decision support.

**Figure 4.**
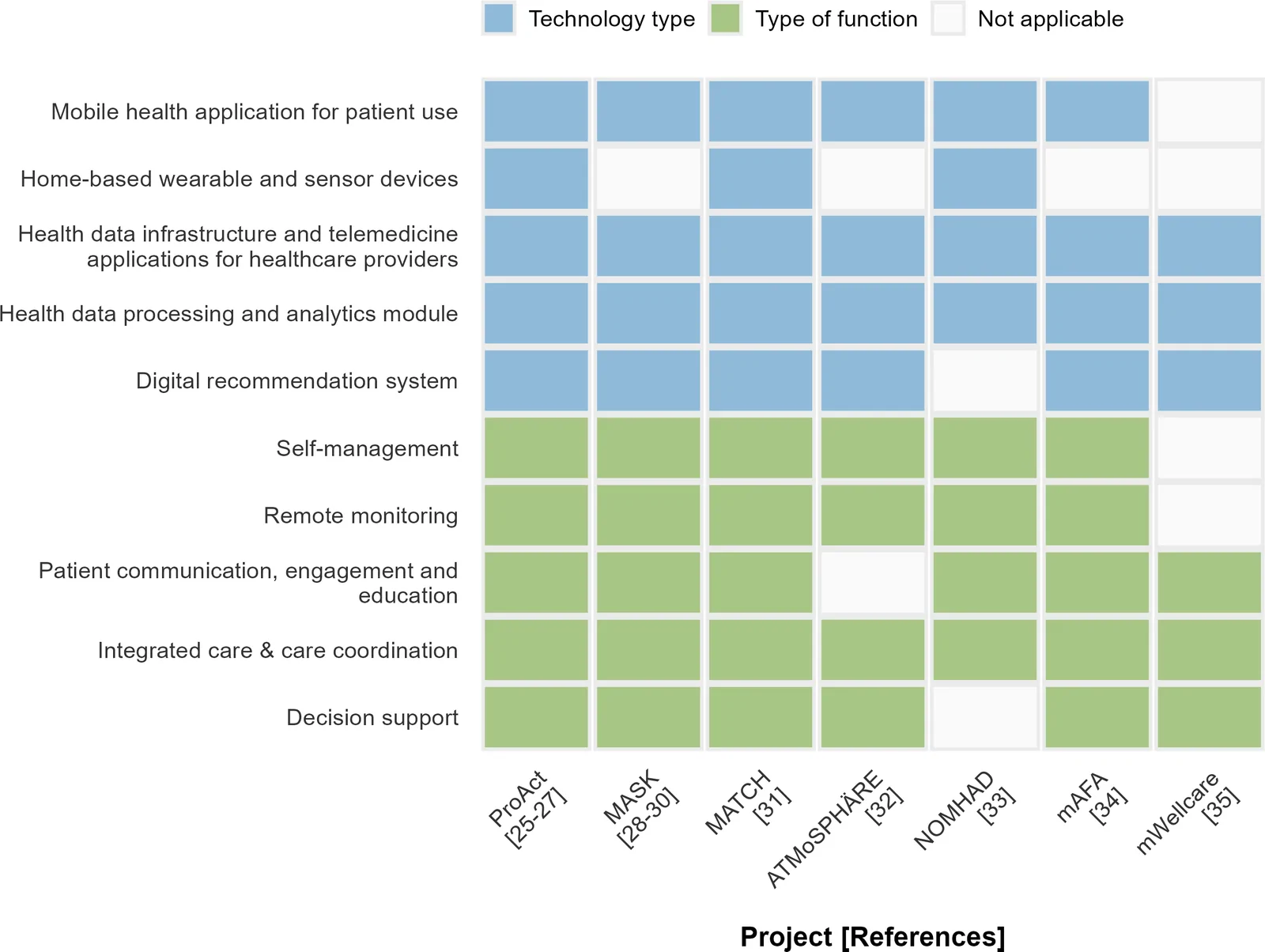
Characteristics of included projects (n=7): digital health technology types and functionalities for managing disease interactions reported in studies (N=11) on digital health solutions accounting for disease interactions in multimorbidity (2014‐2024) [25-35]. ATMoSPHÄRE: Autonomy despite Multimorbidity in Saxony through Patient Empowerment: Holistic Care for Older Adults via the Networking of All Regional Facilities and Service Providers.; CHD: coronary heart disease; CHF: congestive heart failure; COPD: chronic obstructive pulmonary disease; mAFA: Mobile Atrial Fibrillation Application; MASK: Mobile Airways Sentinel Network; MATCH: Multimorbidities Managing Technology for Healthcare; mWellcare: an integrated mHealth System for the Prevention and Care of Chronic Disease; NOMHAD: Novel Model for Health Care Delivery; ProACT: Integrated Technology Systems for Proactive Patient-Centered Care.

The most frequently reported technology categories were health data infrastructure and telemedicine applications for providers, followed by mHealth applications for patients and DRSs (Figure 4). Technologies related to mHealth for patients, clinical recommendation systems, and wearables were also commonly represented across the dataset. Notably, ProACT and MATCH reported the widest range of technology categories. In contrast, mWellcare focused on fewer technology categories, primarily emphasizing digital recommendation and telemedicine without involving patients through a mobile app. Regarding functions, integrated care and care coordination was the only functional category represented across all included projects, underlining its role as a fundamental component in managing patients with multimorbidities. The other functional categories were represented with nearly equal frequency across the dataset (Figure 4).

**Figure 5.**
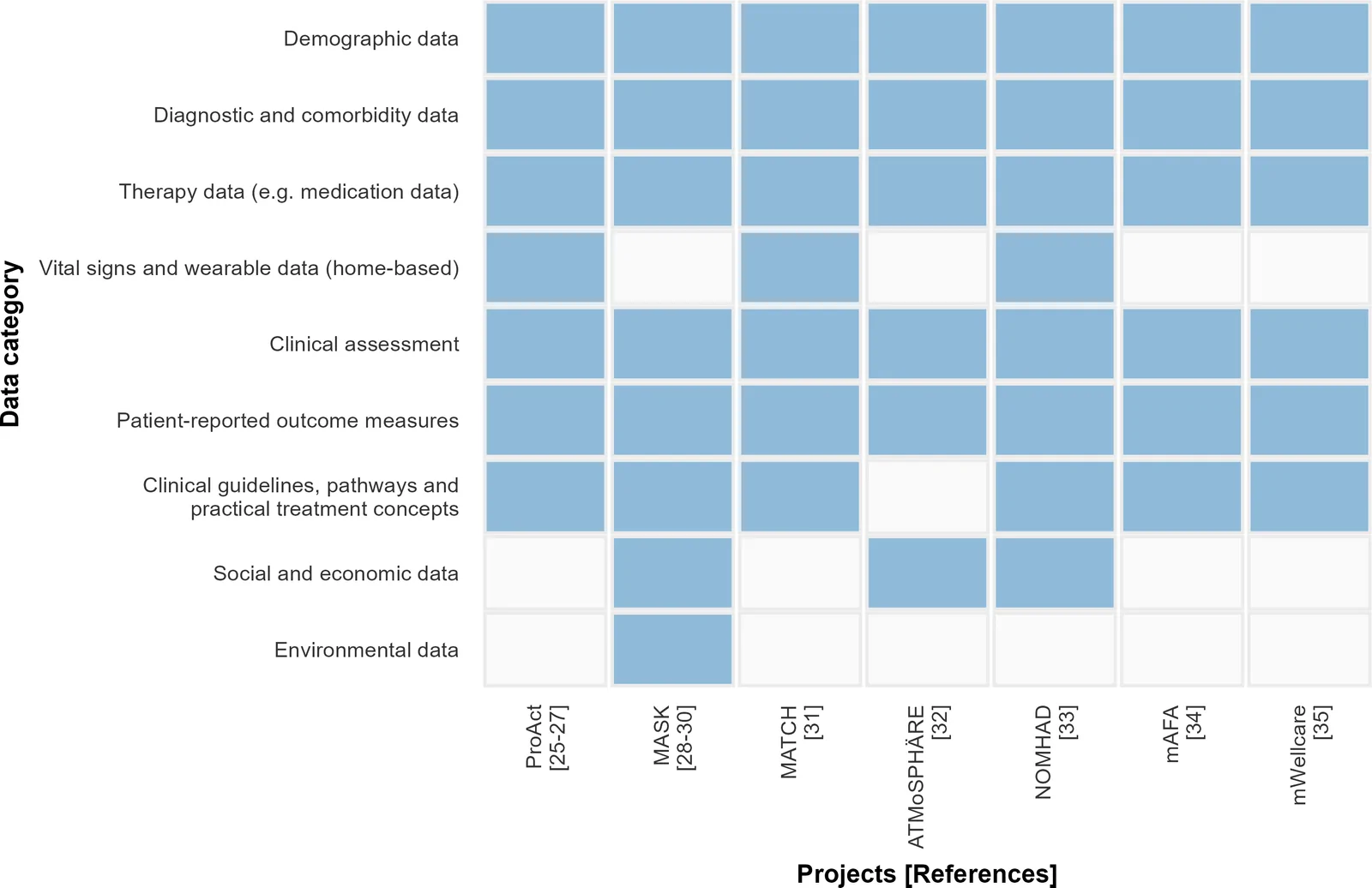
Characteristics of included projects (n=7): extracted data categories reported in studies (N=11) on digital health solutions accounting for disease interactions in multimorbidity management (2014‐2024) [25-35]. ATMoSPHÄRE: Autonomie trotz Multimorbidität in Sachsen durch Patientenempowerment, Holistische Versorgung für Ältere mit Vernetzung aller Regionalen Einrichtungen und Dienstleister; CHD: coronary heart disease; CHF: congestive heart failure; COPD: chronic obstructive pulmonary disease; mAFA: Mobile Atrial Fibrillation Application; MASK: Mobile Airways Sentinel Network; MATCH: Multimorbidities Managing Technology for Healthcare; mWellcare: An integrated mHealth System for the Prevention and Care of Chronic Disease; NOMHAD: Novel Model for Health Care Delivery; ProACT: Integrated Technology Systems for Proactive Patient-Centered Care.

In addition to technologies and functionalities, the analysis identified the main data categories captured across the reviewed projects (Figure 5). The most commonly reported categories include demographic data, diagnostic and comorbidity data, therapy data, and clinical assessment. Following closely, vital signs and wearable data collected in home-based settings, patient-reported outcomes, as well as clinical guidelines along with practical treatment concepts were mentioned in the vast majority of projects. Some projects also consider social and economic factors (eg, social support network and work productivity), which are often relevant in the context of multimorbidity. MASK also integrates environmental data, such as pollen occurrence in regions, providing a holistic view of patient health within the living environment.

### Multimorbidity-Aware System Characteristics and Disease Interactions

The projects were classified based on their alignment with multimorbidity-aware system characteristics. While existing frameworks [37] highlight the need for structured analysis, they focus on clinical reasoning rather than technical system functionalities. We developed a classification scheme focusing on the functional depth of interventions. This categorization allows us to differentiate systems by their level of support, ranging from passive information display to active, prescriptive guidance. Aggregation of multimorbidity-related data is considered a prerequisite and therefore excluded from the classification.

1. Multimorbidity-aware analytics: Analytical methods designed to detect multimorbidity-related patterns, generate risk profiles for patients with multiple chronic conditions, and model potential interactions between diseases.
2. Multimorbidity-aware dashboard and alerts: Visualization and alerting tools tailored to represent the complexity of the state of a patient with multimorbidity. They generate context-sensitive alerts (eg, conflicts and risks) and highlight critical clinical issues (eg, overall risk indicator [ORI]) to assist health care professionals in making individual decisions.
3. Multimorbidity-aware digital recommendation: DRSs (eg, CDSSs) that integrate formal concepts (eg, multiple clinical guidelines), resolve conflicts between disease-specific recommendations, and generate individualized recommendations.

While the classification of multimorbidity-aware system characteristics defines how a system supports clinical decision-making, its practical impact is determined by what specific clinical complexities are being addressed. To provide a holistic view of the digital health interventions, we complement this classification with a clinical dimension centered on disease interaction.

The conceptualization of “disease interactions” in multimorbidity is not uniformly defined in the literature but is discussed across several complementary perspectives. Prior work has highlighted biological interdependencies between coexisting conditions, challenges arising from competing treatment recommendations and polypharmacy, as well as the cumulative symptom burden experienced by patients and the need for coordinated, integrated care processes [5,6,8,11,13]. In addition, research on multimorbidity-oriented clinical decision support emphasizes the importance of integrating multiple data sources and care pathways when managing patients with complex chronic conditions [37].

Building on these perspectives, we developed an analytical framework to categorize how digital health interventions operationalize disease interactions. We distinguish four types of interactions: (1) pathophysiological interactions, referring to biological interdependencies between diseases; (2) treatment-related interactions, including conflicts or synergies between therapies or clinical guidelines; (3) symptom and patient-reported interactions, capturing overlapping or interacting symptoms and patient-reported outcomes; and (4) care process interactions, referring to coordination, prioritization, and integration of care activities across conditions.

**Figure 6.**
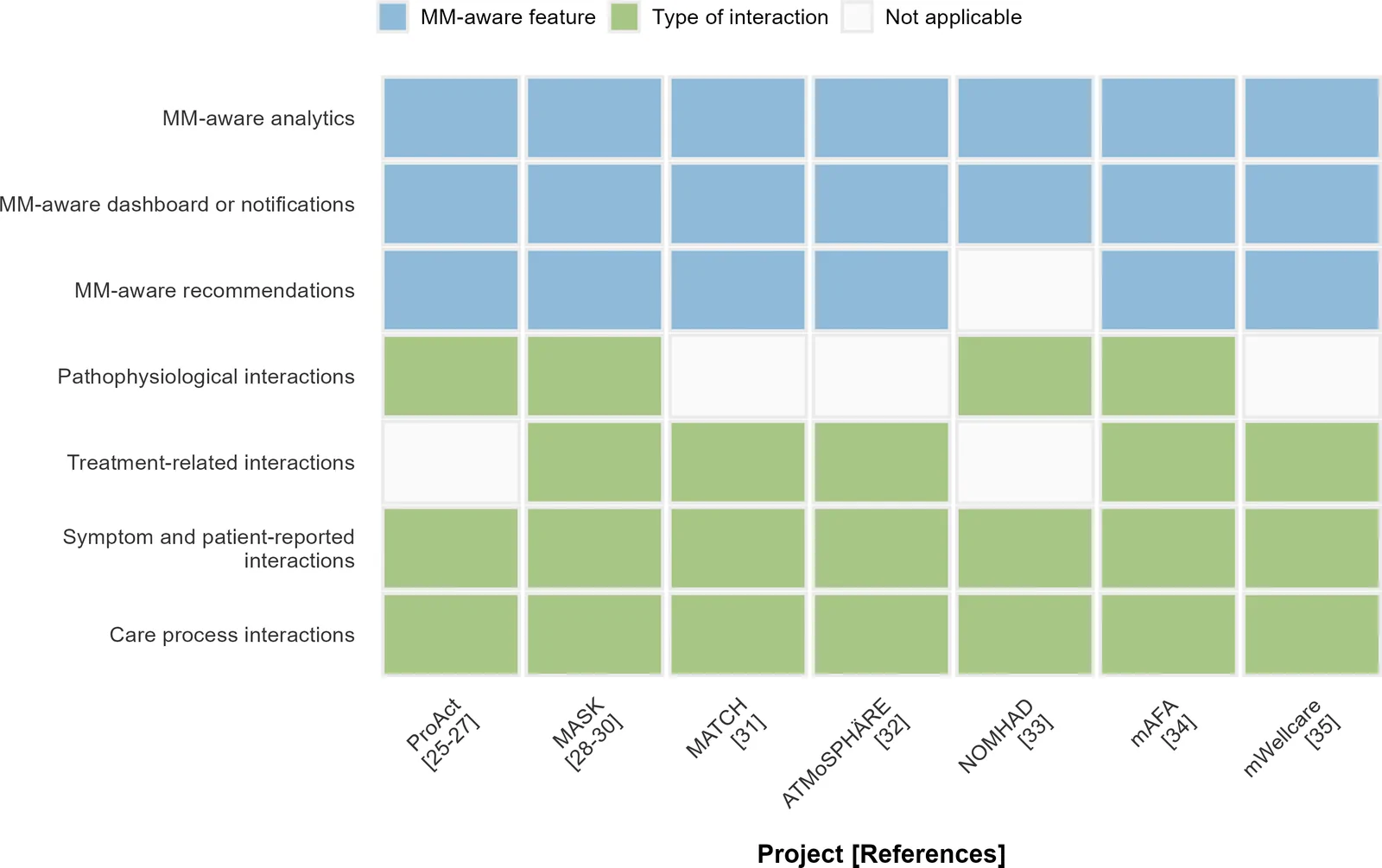
Characteristics of included projects (n=7): functional depth of multimorbidity-aware features and targeted disease interaction categories reported in studies (N=11) on digital health solutions accounting for disease interactions in multimorbidity (2014‐2024) [25-35]. ATMoSPHÄRE: Autonomy despite Multimorbidity in Saxony through Patient Empowerment: Holistic Care for Older Adults via the Networking of All Regional Facilities and Service Providers.; CHD: coronary heart disease; CHF: congestive heart failure; COPD: chronic obstructive pulmonary disease; mAFA: Mobile Atrial Fibrillation Application; MM: multimorbidity; MASK: Mobile Airways Sentinel Network; MATCH: Multimorbidities Managing Technology for Healthcare; mWellcare: An integrated mHealth System for the Prevention and Care of Chronic Disease; NOMHAD: Novel Model for Health Care Delivery; ProACT: Integrated Technology Systems for Proactive Patient-Centered Care.

The analysis of the included projects reveals varying degrees of multimorbidity awareness, ranging from basic data analysis to advanced decision support (Figure 6). Regarding multimorbidity-aware system characteristics, systems like NOMHAD and mWellcare focus on integrated data analysis and risk stratification, exemplified by NOMHAD’s ORI. A higher level of functional depth is reported in ProACT, which uses inferential Bayesian Network (BN) modeling to identify latent biological variables. Similarly, MASK achieves depth by operationalizing the United Airway concept, using systematic pattern recognition to capture the physiological interplay between allergic conditions. Furthermore, projects like mAFA, MATCH, and ATMoSPHÄRE implement prescriptive decision support, using the ABC pathway (A: anticoagulation or avoid stroke; B: better symptom control; and C: cardiovascular disease and comorbidity management) or specialized decision support systems (DSSs) to provide structured clinical guidance. Unlike systems that actively suggest therapy adjustments, NOMHAD acts primarily as a risk-monitoring tool.

The interplay between conditions is operationalized within the projects through different interaction categories. Pathophysiological integration is explicitly addressed in ProACT and MASK, which model biological dependencies between conditions. In contrast, systems like MATCH and ATMoSPHÄRE do not explicitly model these biological synergies. Instead, they manage clinical consequences by reconciling guideline conflicts or integrating multidimensional geriatric assessments to monitor functional decline. MATCH and mWellcare use symptom-based integration by monitoring overlapping clinical markers, such as dyspnea, to detect exacerbations without requiring deep biological modeling.

Treatment-related harmonization is a key feature in ATMoSPHÄRE and mAFA, where conflicting clinical guidelines are reconciled by prioritizing holistic, patient-centered goals. In comparison, while NOMHAD identifies risks, the study does not report any automated tools to adjust treatments. Therefore, based on the published data, clinicians must still resolve therapy conflicts themselves. While ProACT provides holistic self-management support, its treatment-related interactions are primarily focused on harmonizing behavioral goals (eg, activity levels) rather than providing automated logic to resolve conflicting clinical guidelines or pharmacological therapies. Finally, all analyzed projects demonstrate strong care process integration, using integrated dashboards or recommendations to support both patient-health care provider communication and interprofessional coordination among the various health care stakeholders involved. The full details and technical justifications of categorization for each project are provided in Multimedia Appendix 5.

### Target Groups

**Figure 7.**
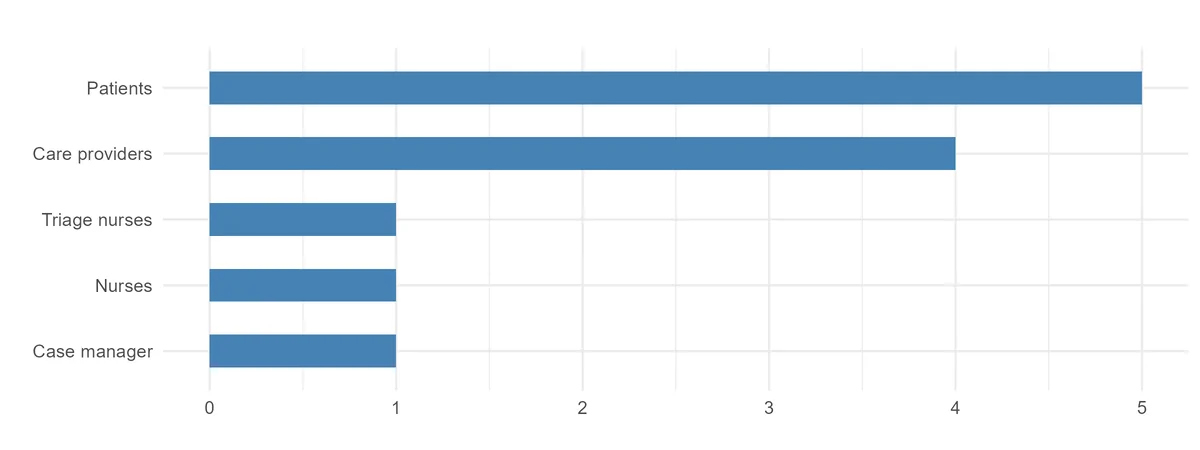
Target groups of the included studies (N=11; 2014‐2024) [25-35].

The majority of the projects (n=5) are primarily designed for patients, aiming to enhance patient empowerment and promote effective self-management of their health (Figure 7). In addition, four projects specifically target care providers, underscoring the importance of a multiperspective approach in managing multimorbidity. Within this framework, nurses, triage nurses, and case managers frequently emerge as pivotal organizational actors who facilitate coordination and provide ongoing support. Their involvement reflects a model of care that integrates professional guidance with patient autonomy.

### Evaluation Methods

Of the 11 included studies, nine [25-27,29,31-35] reported an evaluation phase, while the remaining two [28,30] focused on describing the concept and implementation phase. These nine studies varied in terms of design, population, intervention type, and outcome measurement. The most frequently used study design was the proof-of-concept (PoC) trials (n=3), followed by randomized controlled trials (RCTs; n=2) and interventional studies (single-arm design), observational studies, mixed method designs, and pilot studies with one mention each. Both RCTs used cluster randomization approaches to allocate participants at the health center level.

The sample sizes of the studies involving participants (n=9) ranged from eight in ATMoSPHÄRE (2021) to 8504 individuals drawn from The Irish Longitudinal Study on Ageing (TILDA) cohort study used in ProACT (2018), with a median sample size of 120 participants. In total, these studies involved 19,036 individuals, including patients and health care professionals.

Participant demographics across studies consistently reflected an older population except for MASK 2018 (12-92 years; mean 39, SD 16.5 years). Mean participant ages ranged within the other studies from 55.1 years (mWellcare 2019) to 74.2 years (ProACT studies), with several studies reporting SDs of 6 to 13 years. Gender distributions varied: in the mAFA trial, 33.4% of the intervention group were female compared to 41.9% in the control group; in the NOMHAD study, 87% of participants were male. Studies consistently included participants with at least two chronic conditions.

Only two studies [34,35] used control groups. The mAFA-II and mWellcare trials compared outcomes between intervention and control clusters, whereas the remaining studies were single-arm or observational in nature and focused either on feasibility, system development, or user experience.

Outcome measures were diverse. Clinical outcomes included change in systolic blood pressure, hemoglobin A_1c_ (HbA_1c_) levels, COPD-related quality-of-life scores, and hospitalization rates. Process and behavioral outcomes were also widely reported, such as daily adherence rates to symptom diaries, medication adherence, and digital literacy assessments. User engagement metrics (eg, number of app logins and completion of readings) and usability measures (eg, questionnaire-based satisfaction) were reported in studies such as ProACT 2021 [26] and NOMHAD 2022 [33]. Some studies (eg, ProACT 2018 [25]) focused solely on algorithmic prediction accuracy, reporting performance metrics like Brier scores.

Duration of follow-up or observation ranged from 3 months (eg, NOMHAD 2022 [33]: mean 78.7 days) to 12 months or longer (eg, mAFA-II [34], ProACT [25-27], and MASK 2018 [29]). Most RCTs and PoC trials included clearly defined timelines with multiple data collection points, while observational studies (eg, ATMoSPHÄRE 2021 [32]) were either cross-sectional or did not report exact follow-up durations.

### Outcome

The reported outcomes clustered into 4 main areas: user engagement and adherence, usability and implementation, technical performance and user experience, and clinical effectiveness (Table 2).

**Table 2.** Focus and findings of the evaluations reported in the included studies (N=11; 2014‐2024).

Focus area	Projects	Findings
User engagement and adherence	ProACT^a^ [26,27] MATCH^b^ [31]	Sustained engagement, moderate adherence, improved patient confidence
Usability and implementation	^c^ATMoSPHÄRE [32], MASK^d^ [29]	Usable systems aiding GPs^e^, real-world guideline integration, support for allergy care implementation
Technical performance and UX^f^	NOMHAD^g^ [33], ProACT [25]	High algorithmic accuracy and patient satisfaction, demonstrated model accuracy using population health data
Clinical outcomes	mAFA-II^h^ [34], mWellcare^i^ [35]	mAFA-II showed reduced adverse events; mWellcare showed no significant effect

a ProACT: Integrated Technology Systems for Proactive Patient-Centered Care.

b MATCH: Multimorbidities Managing Technology for Healthcare.

c ATMoSPHÄRE: Autonomy despite Multimorbidity in Saxony through Patient Empowerment: Holistic Care for Older Adults via the Networking of All Regional Facilities and Service Providers.

d MASK: Mobile Airways Sentinel Network.

e GP: general practitioner.

f UX: user experience.

g NOMHAD: Novel Model for Health Care Delivery.

h mAFA: Mobile Atrial Fibrillation Application.

i mWellcare: mHealth and Wellness Care.

Studies evaluating patient engagement generally reported sustained use of digital interventions, moderate adherence, and increased confidence in self-management, particularly when technological support was complemented by human assistance. Evaluations of usability and implementation indicated that digital tools could be successfully integrated into routine care and were generally perceived as useful by health care professionals. Studies focusing on technical performance reported high levels of patient satisfaction and promising algorithmic accuracy, highlighting the potential of data-driven approaches for multimorbidity management. Evidence regarding clinical effectiveness was limited and mixed. While the mAFA-II trial demonstrated significant improvements in clinical outcomes, the mWellcare study found no significant intervention effects.

### Synthesis of Results

#### Overview

In the following section, findings are synthesized according to the research objectives to ensure a coherent and structured overview of the evidence.

#### Technology-Enabled Care for Multimorbidity

The included studies describe integrated, multimodal digital systems for multimorbid care rather than isolated tools. On the patient side, mHealth apps are frequently combined with wearable sensors to enable self-management, continuous symptom tracking, and remote monitoring. For health care providers, data infrastructures and telemedicine platforms support care coordination and information management. These provider solutions often incorporate analytics to transform patient data into actionable insights, such as risk indicators and multimorbidity patterns. While some systems only provide basic dashboards and alerts, others use decision support tools and decision recommendation systems to offer personalized, evidence-based recommendations for complex patient profiles.

#### Addressing Disease Interactions in Intervention Design

The included studies show differences in how multimorbidity-aware functionality is implemented across systems. Some approaches focus on analytics that identify multimorbidity-related patterns, generate risk profiles, and, in certain cases, explore interactions between conditions. Other systems emphasize decision support through context-sensitive dashboards and alerting mechanisms that highlight clinically relevant risks and support decision-making in complex patient situations. In more advanced cases, interventions provide prescriptive decision support by integrating clinical guidelines, resolving potential conflicts, and generating individualized recommendations.

Regarding disease interactions, most interventions focused on symptom-level and patient-reported interactions, using composite scores, symptom diaries, or patient-reported outcome measures to capture cross-disease burden. Care process interactions were also frequently addressed through dashboards, risk indicators, and coordinated care workflows supporting prioritization and decision-making across conditions. In contrast, only a few interventions incorporated more advanced pathophysiological modeling, such as BN-based analytics in ProACT, or systematically addressed treatment-related interactions through guideline-based decision support. Overall, current digital health interventions tend to aggregate and relate multimorbidity data, but they rarely operationalize deeper disease interdependencies or explicitly reconcile conflicting treatment recommendations.

#### Concepts for Integrated, Personalized Multimorbidity Care

**Figure 8.**
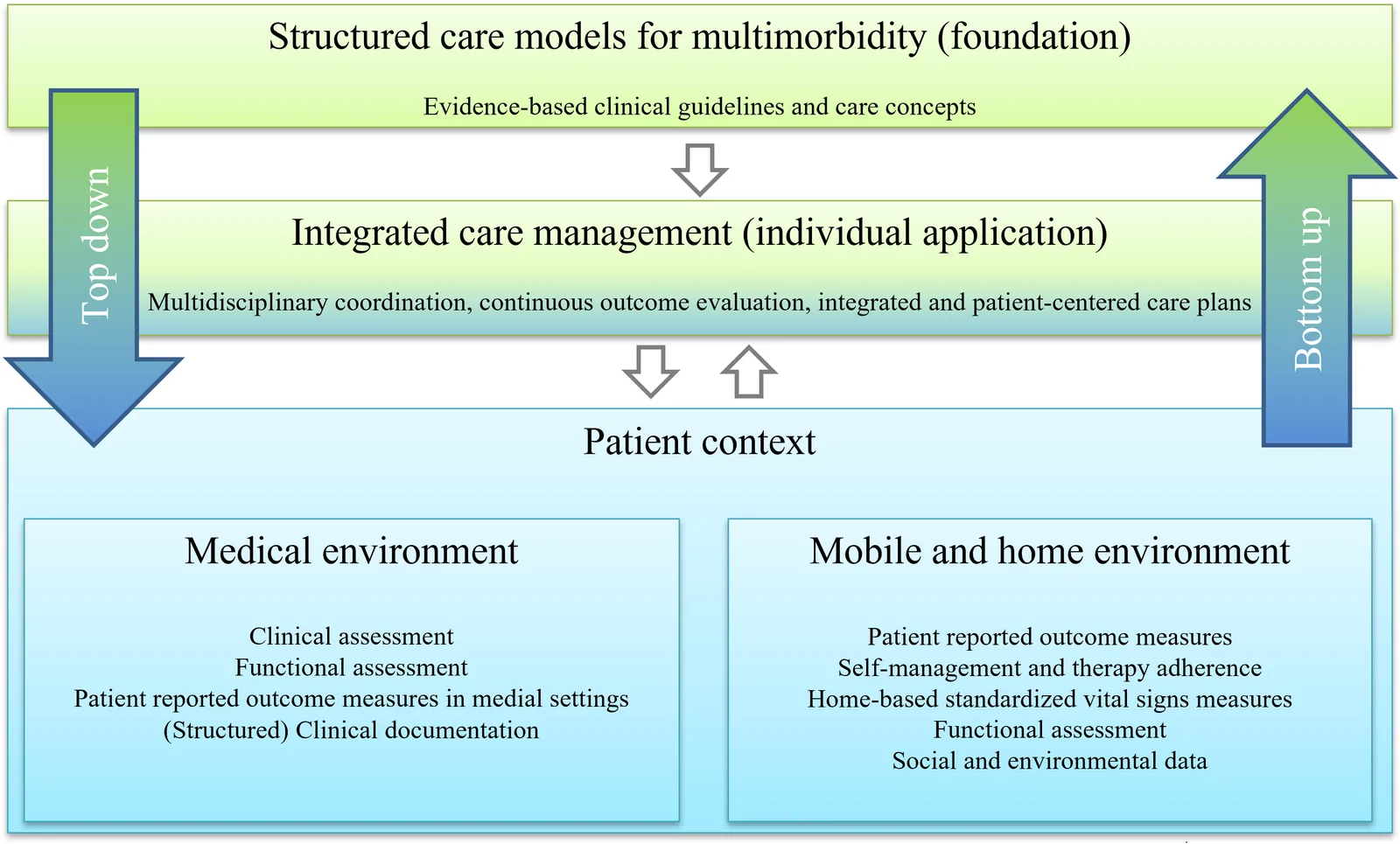
Conceptual overview of multimorbidity care approaches: Synthesis of components for managing disease interactions across included projects (n=7) based on a review of 11 included studies (2014‐2024). [25-35].

The involved studies reflect a multidimensional approach to managing multimorbidity, integrating top-down, guideline-driven strategies and bottom-up, patient-centered data-driven models. The findings can be organized into three complementary domains (Figure 8): (1) structured care models for multimorbidity, (2) integrated care management, and (3) standardized documentation of patient health profiles.

The first domain is based on clinical guidelines covering both disease-specific recommendations and multimorbidity-oriented frameworks. The ABC pathway and the Allergic Rhinitis and its impact on Asthma (ARIA) 2015 guideline demonstrate this by promoting holistic, integrated care across coexisting conditions. Embedding such guidelines into digital interventions allows for automated, evidence-based recommendations for complex patient profiles. Integrated care management relies on structured programs that coordinate roles, tools, and care plans across providers. Defined roles (triage nurses and case managers) guide patients through complex treatment pathways, while digital interventions help translate evidence-based protocols into clinical practice.

Standardized documentation uses validated scoring and assessment instruments to describe complex patient profiles, identify individual priorities, and support dynamic adjustment of care. These include disease-specific risk scores such as Congestive heart failure, Hypertension, Age, Diabetes mellitus, Stroke/transient ischemic attack/thromboembolism, Vascular disease, Age, Sex category (CHA₂DS₂); Hypertension, Abnormal renal/liver function, Stroke, Bleeding history or predisposition, Labile international normalized ratio, Elderly, Drugs/alcohol concomitantly (HAS-BLED); and Sex, Age, Medical history, Treatment (interacting drugs), Tobacco use, Race (non-Caucasian; SAMe-TT₂R₂); SAMe-TT₂R₂ in mAFA-II; the ORI composite index in NOMHAD; and geriatric assessments such as the Instrumental Activities of Daily Living (IADL) scale, Mini-Mental State Examination, and Timed Up-and-Go in ATMoSPHÄRE. Patient-reported outcomes including symptom diaries, quality-of-life questionnaires, and standardized tools such as the International Physical Activity Questionnaire (IPAQ) and Control of Allergic Rhinitis and Asthma Test (CARAT) support active self-monitoring alongside clinician-administered assessments. Continuous vital sign tracking via wearables and visual analog scales (VAS) in MASK further extend data collection. Together, these instruments support automated risk stratification and feed analytical modules to enable adaptive, need-based treatment planning.

#### Frequently Targeted Diseases and Recurring Multimorbidity Clusters

The analyzed studies targeted various multimorbidity clusters, including cardiometabolic multimorbidity, cardiorespiratory multimorbidity, and allergy-asthma cluster. These clusters predominantly consist of chronic conditions, rather than acute illness, thus addressing long-term treatment and management. The multimorbidity clusters are characterized by overlapping symptoms and complex disease interactions between conditions, which complicate treatment. These challenges are addressed by digital solutions examined in this review. For example, we identified three projects (ProAct, MATCH, and NOMHAD) that focus on the cooccurrence of COPD and CHF, partly in combination with other diseases, highlighting the importance of managing overlapping symptoms, shared risk factors, therapeutic overlaps, and pathophysiological interactions of these two diseases. There is only one project that targets a multimorbidity cluster (allergy-asthma) also relevant to the younger population. Apart from this project, we found others focusing also on younger populations, which we excluded because the digital solutions address only a single disease in patients with multimorbidities. However, there is clear evidence that multimorbidity also affects younger populations (eg, addiction-depression cluster), but multimorbidity-focused approaches for the younger population remain insufficiently addressed in current digital health solutions.

#### Reported Effectiveness and Implementation Outcomes of Multidisease Approaches

The reviewed studies demonstrate that digital health interventions targeting patients with multiple chronic conditions can be both feasible to implement and potentially effective. Successful implementation was generally associated with high usability, adaptability to clinical settings, and the integration of personal support mechanisms.

Evidence suggests that effectiveness in multidisease contexts depends not only on the technological capabilities of the intervention but also on how well it is embedded within broader care pathways. Interventions that combined digital tools with human support, such as health professionals assisting with data interpretation, tended to show stronger engagement and sustained use over time.

While one reported measurable clinical benefit, another highlighted implementation success without clear clinical superiority over usual care. This variability points to the importance of context, user engagement, and implementation fidelity in shaping outcomes.

Overall, the findings support the potential of multidisease digital approaches, especially when they are designed to be user-centered, support hybrid care models, and align with existing health care infrastructures. However, further research is needed to understand the conditions under which these interventions deliver consistent clinical value at scale.

#### Challenges and Barriers to the Real-World Application of Interventions

While several studies mention challenges, these are often described only in general terms or focus on different aspects, without directly addressing our specific research question. Overall, the field appears to be in its early stages, with most of the existing literature limited to feasibility or explorative research. As such, critical steps toward the broader implementation and scaling of these interventions remain largely unaddressed. Consequently, our guiding question regarding the reported challenges, limitations, or barriers affecting the practical application, scalability, or sustainability of these interventions in real-world settings cannot yet be adequately answered, as current research has not progressed to this stage.

## Discussion

### Summary of Evidence

This scoping review identified only a small number of digital health interventions that explicitly address multimorbidity as an interconnected phenomenon rather than a collection of separate chronic diseases. Although several interventions supported patients with multiple chronic conditions, only a few implemented mechanisms to model disease interactions or reconcile competing treatment recommendations.

This reflects broader challenges in multimorbidity care, where health care structures and clinical guidelines continue to be organized around individual diseases [5,6,8,11,13]. Several factors may explain why multimorbidity-aware digital interventions remain limited in practice. First, health care systems and clinical guidelines are still predominantly organized around single diseases, making integrated digital modeling of multiple conditions inherently difficult [5,6,8,13]. Second, multimorbidity introduces substantial technical and clinical complexity, including overlapping symptoms, competing treatment recommendations, heterogeneous patient trajectories, and high data integration requirements [8,11,37]. Third, the development and evaluation of such systems often require interdisciplinary collaboration across clinical, technical, and organizational domains, which may limit scalability and implementation in routine care settings [12,13]. Together, these challenges may contribute to the predominance of symptom-level monitoring and care coordination approaches over more advanced multimorbidity modeling strategies.

Although theoretical models for integrated multimorbidity care already exist, their digital translation remains limited. Established clinical and public health frameworks, including patient-centered care models, provide conceptual direction, yet current eHealth systems rarely operationalize them in practice [8,12,38]. Many interventions aggregate information across conditions without explicitly modeling their interdependencies or supporting integrated therapeutic decision-making. Similar fragmentation has also been described in traditional care settings, where established links between diseases, such as diabetes and periodontitis, are often recognized clinically but not jointly managed [39].

Beyond summarizing the available evidence, this review provides a structured perspective on multimorbidity-aware digital systems by distinguishing both the functional depth of technological support (analytics, dashboards, and recommendation systems) and the types of disease interactions addressed (pathophysiological, treatment-related, symptom-related, and care-process interactions). This dual perspective may support future development and evaluation of multimorbidity-oriented digital interventions by providing a common framework for describing system capabilities.

The included studies highlight important facilitators for successful implementation. Interventions combining remote monitoring, self-management support, data analytics, and structured care roles (eg, triage nurses and case managers) demonstrated that successful multimorbidity care depends not only on technical functionalities but also on organizational integration and human support [25-33]. However, even these interventions primarily addressed symptom-level and care-process integration rather than deeper pathophysiological or treatment-related interactions.

Overall, digital health for multimorbidity is still in an early developmental phase, in which conceptual models, technical capabilities, and care structures are not yet fully aligned. Most evidence therefore remains limited to feasibility, usability, and implementation outcomes rather than clinical effectiveness. Future research should focus on interoperable multimorbidity-aware infrastructures that integrate heterogeneous patient data, support coordinated care pathways, and investigate how disease interactions can be represented computationally and translated into transparent, clinically actionable decision support. In addition, clinical effectiveness needs to be systematically evaluated to build stronger evidence.

### Limitations

This review has several limitations that should be acknowledged. First, the literature search was limited to two databases (PubMed and CINAHL) and to publications in English and German. Although this may have resulted in relevant studies being missed, backward reference screening of included studies and relevant reviews identified only one additional eligible study, suggesting that the risk of substantial omission is limited.

Second, this review deliberately applied strict eligibility criteria by including only interventions that explicitly addressed interactions between coexisting conditions. Consequently, digital health interventions targeting multimorbid populations but managing conditions independently, as well as user-centered design studies, technical development papers, and conceptual frameworks without implementation or evaluation, were excluded. The findings should therefore be interpreted as representing a specific subset of multimorbidity-oriented digital interventions rather than the broader landscape of digital health for multimorbid populations.

Third, only 11 studies [25-35] met the strict inclusion criteria. While this reflects the emerging state of research in multimorbidity-aware digital health, it also limits the generalizability of the findings. In addition, the considerable heterogeneity of study designs, intervention types, and reported outcomes precluded formal comparisons of effectiveness.

Finally, the analytical framework developed in this review to classify multimorbidity-aware system functionalities and disease interaction types was derived inductively from the included studies and informed by existing conceptual literature. Although it provides a structured perspective for describing and comparing multimorbidity-oriented digital interventions, it has not yet been externally validated and should therefore be regarded as a conceptual framework rather than an established taxonomy.

### Conclusions

Advancing digital health for multimorbidity will require a transition from disease-oriented technologies toward interoperable, patient-centered systems capable of integrating heterogeneous clinical, behavioral, and contextual data across conditions. Beyond technological innovation, successful implementation will depend on the alignment of digital infrastructures with integrated care models, multidisciplinary workflows, and supportive health care policies.

As multimorbidity increasingly becomes the dominant reality of chronic care, the development of multimorbidity-aware digital systems may become a key prerequisite for scalable and sustainable health care delivery.

## Supplementary material

10.2196/88021 Multimedia Appendix 1 Final search strings for each database.

10.2196/88021 Multimedia Appendix 2 Detailed overview of data items for data extraction.

10.2196/88021 Multimedia Appendix 3 Study-specific data extraction (study design, sample size, and outcomes).

10.2196/88021 Multimedia Appendix 4 Data extraction on project level (addressed diseases, target group, technologies, functionalities, and data categories).

10.2196/88021 Multimedia Appendix 5 Reported functional depth and interaction categories of the included projects (n=7, reported in 11 studies, 2014-2024).

10.2196/88021 Checklist 1 PRISMA-ScR Checklist

## Abbreviations

ABC: A: anticoagulation or avoid stroke; B: better symptom control; and C: cardiovascular disease and comorbidity management
AF: atrial fibrillation
ARIA: allergic rhinitis and its impact on asthma
ATMoSPHÄRE: Autonomy despite Multimorbidity in Saxony through Patient Empowerment: Holistic Care for Older Adults via the Networking of All Regional Facilities and Service Providers
BN: Bayesian Network
CARAT: Control of Allergic Rhinitis and Asthma Test
CDSS: clinical decision support system
CHA2DS2: Congestive heart failure, Hypertension, Age, Diabetes mellitus, Stroke/transient ischemic attack/thromboembolism, Vascular disease, Age, Sex category
CHD: coronary heart disease
CHF: congestive heart failure
COPD: chronic obstructive pulmonary disease
DRS: digital recommendation system
DSS: decision support system
HAS-BLED: Hypertension, Abnormal renal/liver function, Stroke, Bleeding history or predisposition, Labile international normalized ratio, Elderly, Drugs/alcohol concomitantly
HbA_1c_: hemoglobin A_1c_
IADL: Instrumental Activities of Daily Living
IPAQ: International Physical Activity Questionnaire
LLM: large language model
mAFA: Mobile Atrial Fibrillation Application
MASK: Mobile Airways Sentinel Network
MATCH: Multimorbidities Managing Technology for Healthcare
mHealth: mobile health
mWellcare: mHealth and Wellness Care
NOMHAD: Novel Model for Health Care Delivery
ORI: overall risk indicator
PCC: Population, Concept, and Context
PoC: proof of concept
PRISMA-S: Preferred Reporting Items for Systematic Reviews and Meta-Analyses literature search extension
PRISMA-ScR: Preferred Reporting Items for Systematic Reviews and Meta-Analyses extension for Scoping Reviews
ProACT: Integrated Technology Systems for Proactive Patient-Centered Care
RCT: randomized controlled trial
TILDA: The Irish Longitudinal Study on Ageing
VAS: visual analog scale
WHO: World Health Organization
