# Supplementary material for: eHealth Approaches to Multimorbidity Management: Scoping Review of Interventions and Multidisease Strategies
Source: Online J Public Health Inform. 2026 Sep 8;18:e88021. doi: 10.2196/88021 (PMC13559150; doi:10.2196/88021)
Supplement: Supplementary file 1 [file ojphi-v18-e88021-s001.doc]

The finalized search string applied in PubMed:

(

(

"Mobile Applications"[All Fields]

OR "Telemedicine"[All Fields]

OR "Cellular Phone"[All Fields]

OR "computers, handheld"[MeSH Terms]

OR (

"digital health"[Title/Abstract]

OR "digital intervention*"[Title/Abstract]

OR "e-health"[Title/Abstract]

OR "ehealth"[Title/Abstract]

OR "telehealth"[Title/Abstract]

OR "Telemedicine"[Title/Abstract]

OR (

(

"app"[Title/Abstract]

OR "apps"[Title/Abstract]

OR "application*"[Title/Abstract]

OR "technolog*"[Title/Abstract]

OR "platform*"[Title/Abstract]

OR "computer program*"[Title/Abstract]

OR "software"[Title/Abstract]

)

AND (

"device*"[Title/Abstract]

OR "smartphone*"[Title/Abstract]

OR "phone*"[Title/Abstract]

OR "tablet*"[Title/Abstract]

OR "iphone*"[Title/Abstract]

OR "ipad*"[Title/Abstract]

OR "android"[Title/Abstract]

OR "IOS"[Title/Abstract]

)

)

OR "mobile app*"[Title/Abstract]

OR "mobile technolog*"[Title/Abstract]

OR "mobile device*"[Title/Abstract]

OR "mobile comput*"[Title/Abstract]

)

)

AND (

"Comorbidity"[MeSH Terms]

OR (

"multimorbid*"[Title/Abstract]

OR "multi morbid*"[Title/Abstract]

OR "multidisease"[Title/Abstract]

OR "multi-disease"[Title/Abstract]

OR "cumulative illness"[Title/Abstract]

OR "functional morbidity"[Title/Abstract]

)

)

AND (2014:3000/12/12[pdat])

AND (english[Filter] OR german[Filter])

)

Optimized search string applied in CINAHL:

TX (

"Mobile Applications" OR

"Telemedicine" OR

"Cellular Phone" OR

"Computers, Handheld" OR

"digital health" OR

"digital intervention*" OR

"e-health" OR

ehealth OR

telehealth OR

telemedicine OR

(

(app OR apps OR application* OR technolog* OR platform* OR "computer program*" OR software)

AND

(device* OR smartphone* OR phone* OR tablet* OR iphone* OR ipad* OR android OR IOS)

) OR

"mobile app*" OR

"mobile technolog*" OR

"mobile device*" OR

"mobile comput*"

)

AND

(

"Comorbidity" OR

(comorbid* OR "co-morbid*") OR

(multimorbid* OR "multi-morbid*") OR

(multidisease OR "multi-disease") OR

"cumulative illness" OR

"functional morbidity"

)
