# Supplementary material for: eHealth Approaches to Multimorbidity Management: Scoping Review of Interventions and Multidisease Strategies
Source: Online J Public Health Inform. 2026 Sep 8;18:e88021. doi: 10.2196/88021 (PMC13559150; doi:10.2196/88021)
Supplement: Supplementary file 2 [file ojphi-v18-e88021-s002.doc]

**^Table S1^**^. Detailed overview of data items for data extraction^

| **Item** | **Definition** |
| --- | --- |
| **General Information** | |
| Authors | The names of the authors. |
| Year | Publication year. |
| Title | Title of the paper. |
| Project Name | Name or title of the project or intervention studied, if specified. |
| Country | Country where the study was conducted. |
| **Disease Characteristics** | |
| Diseases | Specific diseases or conditions addressed in the study. |
| Degree of Multimorbidity | Level or extent of multimorbidity reported, such as number of coexisting conditions or severity. |
| **Digital solutions** | |
| Type of Technology | Type of digital or eHealth technology used in the intervention (e.g., mobile health application for patient use, home-based wearable and sensor devices, health data processing and analytics module). |
| Type of Function | Type of functions supported by the eHealth intervention (e. g., self-management, remote monitoring, patient communication, engagement and education). |
| Technological Support of Multimorbidity | Approach to managing multiple chronic conditions from a technical perspective (e.g., multimorbidity-aware analytics, multimorbidity-aware dashboard and/or alerts, multimorbidity-aware decision support). |
| Disease Interaction Type | Categorization of how digital health interventions operationalize disease interactions ((1) pathophysiological interactions, (2) treatment-related interactions, (3) symptom and patient-reported interactions, and (4) care process interactions). |
| Data Categories | Data categories captured in the eHealth intervention (e.g., demographic data, diagnostic and comorbidity data, therapy data, clinical assessment, patient-reported outcomes). |
| Target Group | Intended users or beneficiaries of the intervention (e.g., patients, healthcare providers, nurses). |
| **Evaluation methods** | |
| Study design | Type of study conducted (e.g., randomized controlled trial, pilot study, observational study). |
| Sampling Size | Number of participants included in the study or intervention. |
| Sample Demographics | Key characteristics of the participants such as age, gender, ethnicity, and relevant health status. |
| Intervention Group | Description of the group receiving the eHealth or digital intervention. |
| Control Group | Description of the comparison or control group, if applicable. |
| Outcome Measures | Variables or metrics used to assess the effectiveness or impact of the intervention. |
| Duration of Intervention | Length of time over which the intervention was applied or observed. |
| **Outcome** | |
| Primary Outcome | The main result or endpoint that the study was designed to evaluate. |
| Secondary Outcome | Additional results or endpoints of interest beyond the primary outcome. |
