# Supplementary material for: eHealth Approaches to Multimorbidity Management: Scoping Review of Interventions and Multidisease Strategies
Source: Online J Public Health Inform. 2026 Sep 8;18:e88021. doi: 10.2196/88021 (PMC13559150; doi:10.2196/88021)
Supplement: Supplementary file 3 [file ojphi-v18-e88021-s003.doc]

**Table 1**. Study-specific data extraction (study design, sample size and outcomes).

| **First author, year, project name** | **Develop- ment phase** | **Study design, sample size** | **Sample demographics** | **Intervention group (IG), control group (CG)** | **Duration of intervention** | **Outcome measures** | **Primary outcome (PrO), secondary outcome (SeO)** |
| --- | --- | --- | --- | --- | --- | --- | --- |
| Deparis, 2018, ProACT | Evaluation PoC; feasibility, acceptance and satisfaction of patients and professionals, effectiveness | Technical PoC trial, TILDA dataset (8504 individuals) | aged 50 and over; probably mixed sex; conditions: CHD/ CHF, COPD, diabetes | IG: none  CG: none | not reported | Brier score–based predictive accuracy of the Bayesian Network; usability and interpretability via HWProfile UI for multimorbidity prediction and exploration | PrO: Model achieved low-to-moderate Brier scores (0.43–0.65), showing accurate probabilistic predictions even with some uncertainty in parent variables.  SeO: HWProfile UI demonstrated effective visualization and user interaction for probabilistic inference, supporting decision-making and education in multimorbidity care |
| Doyle, 2021, ProACT | Evaluation | PoC trial, 120 participants | • Age: Mean ~74.23 (SD 6.4) in Ireland; ~73.61 (SD 6.49) in Belgium.  • Gender: In Ireland, 60% male; in Belgium, 72% male.  • Conditions: All had ≥2 of the following chronic diseases: diabetes, COPD, chronic heart failure (CHF), chronic heart disease (CHD) | IG: All participants got access to the ProACT platform: includes a tablet / CareApp, connected sensor/devices (blood pressure cuff, weight scale, activity/sleep wearable; plus glucometer for those with diabetes; pulse oximeter for those with COPD), educational content/tips, technical helpdesk, and clinical triage service to respond to alerts  CG: none | 12 months PoC trial. Data collection at 4 time points: baseline (T1), after each 3‐month action research cycle (T2, T3), and a final post‐trial time point (T4) | • Engagement metrics: number of symptom readings per day, daily device usage, how often participants used different app sections (e.g., reading vitals, dashboard, tips, etc.)  • Usability (via validated questionnaires) and user burden (validated instruments)  • Qualitative outcomes: semistructured interviews exploring participant experiences, benefits, facilitators and barriers to engagement; self‐management, health & well‐being | PrO: Of 120 participants with multimorbidity, 93 (77.5%) remained engaged throughout the trial. The maximum number of participants recorded taking measurements on any day during the trial was 48 in Ireland and 60 in Belgium. Participants had an average of 2 daily readings in Ireland and three daily readings in Belgium. This demonstrated sustained engagement with the platform over one year.  SeO: Usability, measured using the System Usability Scale (SUS), remained consistently high across time points (mean SUS scores above 72), No significant increases in user burden were experienced over time (user burden questionnaire that measures the burden across six constructs, including difficulty of use, physical, time and social, mental and emotional, privacy, and financial) |
| Doyle, 2022, ProACT | Proof-of-concept action-research trial with iterative co-design cycles | PoC trial, 120 participants | Ireland: mean age 74.23 ± 6.4 (65–92 years); Belgium: mean age 73.88 ±  6.23 (65–91); two conditions from the following: COPD, CHF, CHD and diabetes; > 65 years | IG: All participants received ProACT platform plus triage nurse support (nurse-led telephone triage responding to alerts from health monitoring devices); no control group  CG: none | one year | Qualitative themes: role and benefits of triage nurses, patient–nurse relationship, tensions like anxiety; user engagement and usability from broader trial | PrO: Improved self-management, reduced alerts and hospital use; Enhanced confidence and reassurance; Some anxiety and alert-threshold mismatches noted.  ScO: Achieved across biopsychosocial, therapeutic alliance, and shared decision-making dimensions; strong rapport, education, holistic support |
| Bousquet, 2015, MASK | Implementation phase using ICT tools and CDSS as part of guideline deployment under EIP (European Innovation Partnership) on Active and Healthy Ageing | No evaluation | - | - | - | - | - |
| Bousquet, 2018, MASK | Evaluation | prospective observational study, 4210 participants | ranged in age from 12 to 92 years (mean ± SD: 39 ± 16.5 years); 2 169 females (51.5%) and 2 041 males (48.5%) | IG: hypothesis-driven approach was used to select groups depending on VAS levels (high level: VAS ≥ 50/100, low level: VAS < 20/100)  CG: none | 1 year | Prevalence and control of the daily allergic multimorbidity (asthma, conjunctivitis, rhinitis) according to recorded daily overall control (global evaluation); impact of multimorbidity on work and the characterization of multimorbid patterns | PrO: Analysis of over 32,000 user-days showed that global symptom burden was significantly associated with combinations of rhinitis, asthma, and conjunctivitis, with 2.9% of days classified as “extreme uncontrolled” (high burden in all three conditions) SeO: Higher symptom burden, especially in the extreme uncontrolled pattern, was associated with significantly greater impairment in daily work ability, with median work-related VAS scores reaching 64 compared to 37 in milder symptom patterns |
| Bousquet, 2018b, MASK | Implementation of real-world evidence approach under guideline-based care | No evaluation | - | - | - | - | - |
| Sloots, 2021, MATCH | Evaluation | Prospective pilot study, 11 participants | patients with both COPD and CHF; mean age of 66.8 ± 2.9 years; Gender (male), n (%): 7 (63.6); moderate (55%, n=6) to severe (45%, n=5) COPD, five (46%) patients had midrange left ventricular function (LVF) and three (27%) patients had reduced LVF; Three (27.3%) patients had ischemic heart disease and two (18.2%) had diabetes mellitus as comorbid diseases. Eight patients (72.7%) had at least one COPD exacerbation and six patients (54.5%) had increased the dose of diuretics because of decompensated CHF in the previous year. Three patients (27.3%) had a low digital health literacy score (eHealth Literacy Scale (eHEALS) <26). Six patients (54.5%) were familiar with using a tablet at home | IG: patients participated in three self-management training sessions. After the first session, patients started to use an eHealth self-management intervention on a tablet for a period of a maximum of four months  CG: none | 4-month | Adherence to the eHealth self-management intervention in patients with both COPD and heart failure.  Inhalation technique and patient-reported measures. | PrO: In total, 1148 (91%) of the daily diaries were completed on the same day by 11 participating patients. Seven patients received a total of 24 advised actions because of increased symptoms of which 11 (46%) were followed-up. Of the 13 (54%) unperformed advised actions, six were “call the case manager”. Adherence to inhaled medication was 98.4%.  SeO: Only 51.9% of inhalations were performed correctly, with short inhalation time being the most common error (79.6%). |
| Borchers, 2021, ATMOSPHÄRE | Prototype proof-of-concept and plausibility testing phase for the DRS | Mixed-method plausibility assessment (Delphi procedure followed by interviews and quantitative comparison via paired-samples Wilcoxon test), 8 GPs, 2 case studies | of the 8 participating GPs in Delphi, 6 female, 2 male; age mean ~50.7 years, SD ~7.8.  • GPs working in primary care with ≥10 years of professional experience.  • Case vignette patients: e.g. Profile 1: male, 81 years, ~12 diagnoses, ~10 prescription drugs; Profile 2: female, 89 years, ~18 diagnoses, ~7 prescription drugs | IG: none  CG: none | not reported; plausibility testing appears to be a one-time evaluation rather than longitudinal | Degree of appropriateness of DRS recommendations as rated by GPs; Statistical comparison between GP and algorithm recommendations using Wilcoxon test.  Usefulness, omissions, acceptability of th DRS. | PrO: First Delphi round revealed a high variability regarding the amount and character of services recommended by the physicians (1 to 10 recommendations, mean = 5.6, sd = 2.8). These professional recommendations converged after consideration of the algorithm-generated recommendations. The number of algorithm-generated recommendations which were judged as appropriate ranged between 7 and 17 of a total of 20 (mean = 11.9, sd = 2.5)  SeO: All four interviewees confirmed in principle that the DRS is of added value to the GPs and the patient care. |
| Chacornac, 2022, NOMHAD | Pilot, single-arm proof-of-concept interventional study | Interventional study (singe-arm design), 23 participants | Mean age: 68.5 (SD 10.4); 20 of 23 were men; most frequent multimorbidity was CHF + diabetes (n=15). Five patients had all 3 diseases (CHF + diabetes + COPD | IG: Participants performed regular self-assessments of their health status according to their chronic diseases. Patients used the tablet to enter their answers to health questionnaires and the devices connected to the tablet to measure vital signs (eg, blood pressure, pulse rate, arterial oxygen saturation, daily blood glucose, body weight depending on their medical condition, and personalized care plan) and transmit them to the NOMHAD Chronic system  CG: none | Mean follow-up: 78.7 days (SD 24.2); range 80–100 days; system was evaluated over 3 months of follow-up in a single group | Technical accuracy (ORI vs raw data recalculation); Satisfaction (patients, nurses, physicians); Adherence and usability | PrO: Technical Performance: The NOMHAD system demonstrated high accuracy in generating Overall Risk Indicators (ORIs) based on patient data. The system's sensitivity was 99.2% (95% CI 98.9–99.4), and specificity was 91.3% (95% CI 87.7–94.1)  SeO: User Experience: Patients: 87% reported satisfaction with the system and felt it helped them better understand and manage their diseases. Nurses and Physicians: Generally satisfied with the system, considering it useful for patient management. |
| Yao, 2021, mAFA | Fully implemented and evaluated as a cluster-randomized controlled trial | Prospective, cluster-randomized clinical trial, 1890 participants | Intervention: mean [SD] age, 72.0 [12.0] years; 278 [33.4%] women; Usual care: mean [SD] age, 72.8 [13.0] years; 443 [41.9%] women; (≥2 comorbidities, including hypertension, coronary artery disease, heart failure [HF], cardiomyopathy, peripheral arterial disease, diabetes, liver or kidney dysfunction, pulmonary disease, prior stroke | IG: Participants receiving the mHealth-supported ABC pathway via the mobile app  CG: Participants receiving usual care as per standard clinical practice | Mean follow-up: intervention group 419 (SD 257) days; usual care 457 (SD 154) days | Reduction of the composite outcome of stroke or thromboembolism, all-cause death, rehospitalization, irrespective of age, sex, and prior stroke.  Individual components and comorbidity-related events. | PrO: The intervention group demonstrated a significant reduction in this composite outcome compared to usual care (HR 0.37; 95% CI, 0.26–0.53; P < .001).  SeO: Rehospitalization: Significantly reduced in the intervention group (HR 0.42; 95% CI, 0.27–0.64; P < .001). Acute Coronary Syndrome, Heart Failure, and Uncontrolled Blood Pressure: Lower rates observed in the intervention group (27 patients [3.2%] vs 145 patients [13.7%]; HR 0.29; 95% CI, 0.19–0.45; P < .001). Subgroup Analyses: Consistently lower hazard ratios for the primary composite outcome and rehospitalization in the intervention group across various subgroups, including age, prior stroke, and sex. |
| Prabhakaran, 2019, mWellcare | Evaluation | Prospective, multicenter, open-label, cluster-randomized controlled trial, 4270 participants | mean age was 55.1±11.0 years, 55.2% were male, 42.9% had higher than primary school education, and 27.1% were employed | IG: Centralized training on the current clinical management guidelines to all physicians  CG: Enhanced Usual Care (training on clinical management guidelines for hypertension and diabetes mellitus for physicians, management charts for these conditions displayed at outpatient clinics, training in managing hypertension and diabetes mellitus for NCD nurses, tablets for nurses (without the mWellcare system) for baseline data collection, lifestyle advice pamphlets (in Hindi and Kannada) by NCD nurses) | 12-month | Between-group differences in mean change (from baseline to 1 year) in SBP and glycated hemoglobin (HbA1c) among participants with hypertension and diabetes mellitus, respectively  Between-group difference in mean change (from baseline to 1 year) in fasting plasma glucose, total cholesterol, predicted 10-year risk of CVD with the recalibrated Framingham risk score, tobacco use, body mass index, and alcohol use | PrO: Systolic Blood Pressure (SBP) and HbA1c: No significant difference between the mWellcare and enhanced usual care groups.  SeO: No differences in fasting blood glucose, total cholesterol, predicted 10-year risk of CVD, body mass index, and tobacco and alcohol use. |

References:

25. Deparis S, Pascale A, Tommasi P, Kotoulas S. An Analytical Method for Multimorbidity Management Using Bayesian Networks. Stud Health Technol Inform. 2018;247(247):820-824. [doi: 10.3233/978-1-61499-852-5-820] [Medline: 29678075]

26. Doyle J, Murphy E, Gavin S, et al. A Digital Platform to Support Self-management of Multiple Chronic Conditions (ProACT): Findings in Relation to Engagement During a One-Year Proof-of-Concept Trial. J Med Internet Res. Dec 15, 2021;23(12):e22672. [doi: 10.2196/22672] [Medline: 34914612]

27. Doyle J, McAleer P, van Leeuwen C, et al. The role of phone-based triage nurses in supporting older adults with multimorbidity to digitally self-manage - Findings from the ProACT proof-of-concept study. Digit Health. 2022;8(8):20552076221131140. [doi: 10.1177/20552076221131140] [Medline: 36238753]

28. Bousquet J, Schunemann HJ, Fonseca J, et al. MACVIA-ARIA Sentinel NetworK for allergic rhinitis (MASK-rhinitis): the new generation guideline implementation. Allergy. Nov 2015;70(11):1372-1392. [doi: 10.1111/all.12686]

29. Bousquet J, Devillier P, Anto JM, et al. Daily allergic multimorbidity in rhinitis using mobile technology: A novel concept of the MASK study. Allergy. Aug 2018;73(8):1622-1631. [doi: ] [Medline: 29569295]

30. Bousquet J, Arnavielhe S, Bedbrook A, et al. MASK 2017: ARIA digitally-enabled, integrated, person-centred care for rhinitis and asthma multimorbidity using real-world-evidence. Clin Transl Allergy. Dec 2018;8(1):45. [doi: 10.1186/s13601-018-0227-6]

31. Sloots J, Bakker M, van der Palen J, et al. Adherence to an eHealth Self-Management Intervention for Patients with Both COPD and Heart Failure: Results of a Pilot Study. Int J Chron Obstruct Pulmon Dis. 2021;16(16):2089-2103. [doi: 10.2147/COPD.S299598] [Medline: 34290502]

32. Borchers P, Piller S, Böhme M, Voigt K, Bergmann A. Need-based care of multi-morbid patients - supporting general practitioners with algorithm-generated recommendations of healthcare services (telemedicine-project ATMoSPHÄRE). BMC Fam Pract. Oct 8, 2021;22(1):198. [doi: 10.1186/s12875-021-01537-2] [Medline: 34625053]

33. Chacornac M, Faoro A, Texereau J, Billoet C, Hominal S. Performance of an eHealth (NOMHAD) System Comprising Telemonitoring, Telenotification, and Telecoaching for Patients With Multimorbidity: Proof-of-Concept Study. JMIR Form Res. Mar 11, 2022;6(3):e32205. [doi: 10.2196/32205] [Medline: 35275068]

34. Yao Y, Guo Y, Lip GYH, et al. The Effects of Implementing a Mobile Health–Technology Supported Pathway on Atrial Fibrillation–Related Adverse Events Among Patients With Multimorbidity. JAMA Netw Open. Dec 1, 2021;4(12):e2140071. [doi: 10.1001/jamanetworkopen.2021.40071]

35. Prabhakaran D, Jha D, Prieto-Merino D, et al. Effectiveness of an mHealth-Based Electronic Decision Support System for Integrated Management of Chronic Conditions in Primary Care: The mWellcare Cluster-Randomized Controlled Trial. Circulation. Jan 15, 2019;139(3):380-391. [doi: 10.1161/CIRCULATIONAHA.118.038192] [Medline: 30586732]
