# Supplementary material for: eHealth Approaches to Multimorbidity Management: Scoping Review of Interventions and Multidisease Strategies
Source: Online J Public Health Inform. 2026 Sep 8;18:e88021. doi: 10.2196/88021 (PMC13559150; doi:10.2196/88021)
Supplement: Supplementary file 4 [file ojphi-v18-e88021-s004.doc]

**Table S1**.

| **Project name, reference** | **Country** | **Diseases** | **Degree of Multimorbidity** | **Type of Technology** | **Type of Function** | **Technological Support of Multimorbidity** | **Disease Interaction Type** | **Data Categories** | **Target group** |
| --- | --- | --- | --- | --- | --- | --- | --- | --- | --- |
| ProACT, [25,26,27] | Ireland; Belgium | mild cognitive impairment; Diabetes; COPD; CHF; CHD | two or more chronic conditions | mobile health application for patient use; home-based wearable and sensor devices; health data infrastructure and telemedicine applications for healthcare providers; health data processing and analytics module; digital recommendation system | self-management; remote monitoring; patient communication, engagement and education; integrated care & care coordination; decision support | multimorbidity- aware analytics; multimorbidity-aware dashboard and/or alerts/ notifications; multimorbidity- aware digital recommendation | pathophysiological interactions, symptom and patient-reported interactions, care Process interactions | demographic data; diagnostic and comorbidity data; therapy data (e.g. medication data); vital signs and wearable data (home-based); clinical assessment; patient-reported outcomes (PROs); clinical guidelines/ pathways and practical treatment concepts | patients; care providers; triage nurses |
| MASK, [28,29,30] | Austria, Australia, Belgium, Brazil, Denmark, Finland, France, Germany, Greece, Italy, Lithuania, Mexico, Netherlands, Poland, Portugal, Spain, Sweden, Switzerland, UK | allergic rhinitis; asthma | two chronic conditions | mobile health application for patient use; health data infrastructure and telemedicine applications for healthcare providers; health data processing and analytics module; digital recommendation system | self-management; remote monitoring; patient communication, engagement and education; integrated care & care coordination; decision support | multimorbidity- aware analytics; multimorbidity-aware dashboard and/or alerts/ notifications; multimorbidity- aware digital recommendation | pathophysiological interactions, treatment-related interactions, symptom and patient-reported interactions, care Process interactions | demographic data; diagnostic and comorbidity data; therapy data (e.g. medication data); clinical assessment; patient-reported outcomes (PROs); clinical guidelines/ pathways and practical treatment concepts; social and economic data; environmental data | patients |
| MATCH, [31] | Nieder- lande | COPD; CHF | two chronic conditions | mobile health application for patient use; home-based wearable and sensor devices; health data infrastructure and telemedicine applications for healthcare providers; health data processing and analytics module; digital recommendation system | self-management; remote monitoring; patient communication, engagement and education; integrated care & care coordination; decision support | multimorbidity- aware analytics; multimorbidity-aware dashboard and/or alerts/ notifications; multimorbidity- aware digital recommendation | symptom and patient-reported interactions, treatment-related interactions, care Process interactions | demographic data; diagnostic and comorbidity data; therapy data (e.g. medication data); vital signs and wearable data (home-based); clinical assessment; patient-reported outcomes (PROs); clinical guidelines/ pathways and practical treatment concepts | patients; care providers; case manager |
| ATMOSPHÄRE, [32] | Germany | not specified / limited | not specified / limited | mobile health application for patient use; health data infrastructure and telemedicine applications for healthcare providers; health data processing and analytics module; digital recommendation system | self-management; remote monitoring; integrated care & care coordination; decision support | multimorbidity- aware analytics; multimorbidity-aware dashboard and/or alerts/ notifications; multimorbidity- aware digital recommendation | symptom and patient-reported interactions, treatment-related interactions, care Process interactions | demographic data; diagnostic and comorbidity data; therapy data (e.g. medication data); patient-reported outcomes (PROs); social and economic data | care providers |
| NOMHAD, [33] | France | CHF; COPD; Diabetes | two or more chronic conditions | mobile health application for patient use; home-based wearable and sensor devices; health data infrastructure and telemedicine applications for healthcare providers; health data processing and analytics module | self-management; remote monitoring; patient communication, engagement and education; integrated care & care coordination | multimorbidity- aware analytics; multimorbidity-aware dashboard and/or alerts/ notifications | pathophysiological interactions, symptom and patient-reported interactions, care Process interactions | demographic data; diagnostic and comorbidity data; therapy data (e.g. medication data); vital signs and wearable data (home-based); clinical assessment; patient-reported outcomes (PROs); clinical guidelines/ pathways and practical treatment concepts; social and economic data | patients |
| mAFA, [34] | China | atrial fibrillation; CHF; Diabetes; prior bleeding; prior ischemic stroke or other thromboembolism; pulmonary disease | three or more chronic conditions | mobile health application for patient use; health data infrastructure and telemedicine applications for healthcare providers; health data processing and analytics module | self-management; remote monitoring; patient communication, engagement and education; integrated care & care coordination; | multimorbidity- aware analytics; multimorbidity-aware dashboard and/or alerts/ notifications | pathophysiological interactions, treatment-related interactions, symptom and patient-reported interactions, care Process interactions | demographic data; diagnostic and comorbidity data; therapy data (e.g. medication data); clinical assessment; patient-reported outcomes (PROs); clinical guidelines/ pathways and practical treatment concepts | patients; care providers |
| mWellcare, [35] | India | alcohol use disorder; CHF; Diabetes; Depression | two chronic conditions | health data infrastructure and telemedicine applications for healthcare providers; health data processing and analytics module; digital recommendation system | patient communication, engagement and education; integrated care & care coordination; decision support | multimorbidity- aware analytics; multimorbidity-aware dashboard and/or alerts/ notifications; multimorbidity- aware digital recommendation | symptom and patient-reported interactions, treatment-related interactions, care Process interactions | demographic data; diagnostic and comorbidity data; therapy data (e.g. medication data); clinical assessment; patient-reported outcomes (PROs); clinical guidelines/ pathways and practical treatment concepts | nurses |

References:

25. Deparis S, Pascale A, Tommasi P, Kotoulas S. An Analytical Method for Multimorbidity Management Using Bayesian Networks. Stud Health Technol Inform. 2018;247(247):820-824. [doi: 10.3233/978-1-61499-852-5-820] [Medline: 29678075]

26. Doyle J, Murphy E, Gavin S, et al. A Digital Platform to Support Self-management of Multiple Chronic Conditions (ProACT): Findings in Relation to Engagement During a One-Year Proof-of-Concept Trial. J Med Internet Res. Dec 15, 2021;23(12):e22672. [doi: 10.2196/22672] [Medline: 34914612]

27. Doyle J, McAleer P, van Leeuwen C, et al. The role of phone-based triage nurses in supporting older adults with multimorbidity to digitally self-manage - Findings from the ProACT proof-of-concept study. Digit Health. 2022;8(8):20552076221131140. [doi: 10.1177/20552076221131140] [Medline: 36238753]

28. Bousquet J, Schunemann HJ, Fonseca J, et al. MACVIA-ARIA Sentinel NetworK for allergic rhinitis (MASK-rhinitis): the new generation guideline implementation. Allergy. Nov 2015;70(11):1372-1392. [doi: 10.1111/all.12686]

29. Bousquet J, Devillier P, Anto JM, et al. Daily allergic multimorbidity in rhinitis using mobile technology: A novel concept of the MASK study. Allergy. Aug 2018;73(8):1622-1631. [doi: ] [Medline: 29569295]

30. Bousquet J, Arnavielhe S, Bedbrook A, et al. MASK 2017: ARIA digitally-enabled, integrated, person-centred care for rhinitis and asthma multimorbidity using real-world-evidence. Clin Transl Allergy. Dec 2018;8(1):45. [doi: 10.1186/s13601-018-0227-6]

31. Sloots J, Bakker M, van der Palen J, et al. Adherence to an eHealth Self-Management Intervention for Patients with Both COPD and Heart Failure: Results of a Pilot Study. Int J Chron Obstruct Pulmon Dis. 2021;16(16):2089-2103. [doi: 10.2147/COPD.S299598] [Medline: 34290502]

32. Borchers P, Piller S, Böhme M, Voigt K, Bergmann A. Need-based care of multi-morbid patients - supporting general practitioners with algorithm-generated recommendations of healthcare services (telemedicine-project ATMoSPHÄRE). BMC Fam Pract. Oct 8, 2021;22(1):198. [doi: 10.1186/s12875-021-01537-2] [Medline: 34625053]

33. Chacornac M, Faoro A, Texereau J, Billoet C, Hominal S. Performance of an eHealth (NOMHAD) System Comprising Telemonitoring, Telenotification, and Telecoaching for Patients With Multimorbidity: Proof-of-Concept Study. JMIR Form Res. Mar 11, 2022;6(3):e32205. [doi: 10.2196/32205] [Medline: 35275068]

34. Yao Y, Guo Y, Lip GYH, et al. The Effects of Implementing a Mobile Health–Technology Supported Pathway on Atrial Fibrillation–Related Adverse Events Among Patients With Multimorbidity. JAMA Netw Open. Dec 1, 2021;4(12):e2140071. [doi: 10.1001/jamanetworkopen.2021.40071]

35. Prabhakaran D, Jha D, Prieto-Merino D, et al. Effectiveness of an mHealth-Based Electronic Decision Support System for Integrated Management of Chronic Conditions in Primary Care: The mWellcare Cluster-Randomized Controlled Trial. Circulation. Jan 15, 2019;139(3):380-391. [doi: 10.1161/CIRCULATIONAHA.118.038192] [Medline: 30586732]
