# Supplementary material for: eHealth Approaches to Multimorbidity Management: Scoping Review of Interventions and Multidisease Strategies
Source: Online J Public Health Inform. 2026 Sep 8;18:e88021. doi: 10.2196/88021 (PMC13559150; doi:10.2196/88021)
Supplement: Supplementary file 5 [file ojphi-v18-e88021-s005.doc]

**Table S1**.

| **Project** | **MM-Aware System Features (Functional Depth)** | **Disease-to-Disease Interaction Categories** |
| --- | --- | --- |
| ProACT [25-27] | ✔️ MM-aware analytics: Uses Bayesian Network (BN) modeling based on sensor data and self-report questionnaires to estimate unobserved variables (e.g., blood pressure) based on new observation inputs. The CareAnalytics module performs analysis on aggregated data, supporting self-management with a holistic profile assessment.  ✔️ MM-aware dashboard and alerts: Condition exacerbation monitoring and dashboards that automatically highlight critical statuses and trigger notifications.  ✔️ MM-aware digital recommendation: Personalized goal recommender for weekly targets and education recommender for status-based training materials. | ✔️ Pathophysiological interactions: Uses holistic BN modeling to link biological variables (e.g., activity vs. blood pressure) into a cross-condition profile.  ❌ Treatment-related interactions: Not explicitly addressed. While the system utilizes goal and education recommenders to harmonize lifestyle targets and self-management training, the studies do not report on automated logic to resolve conflicting pharmacological treatments or clinical guidelines.  ✔️ Symptom and patient-reported interactions: Dashboards monitor the overall condition profile to detect how symptoms from multiple diseases interact or exacerbate each other.  ✔️ Care process interactions: Triage tools and integrated dashboards coordinate cross-condition thresholds and information flow between patients and clinicians. |
| MASK [28-30] | ✔️ MM-aware analytics: Employs pattern recognition and risk calculation through the e-Allergy screening (incorporating various physician-inputted allergy data) and VAS (Visual Analogue Scales) for daily recording of nasal, ocular, and other symptoms. This enables continuous monitoring, trend analysis, and the calculation of composite scores like CARAT (Control of Allergic Rhinitis and Asthma Test) and RAPP (RhinAsthma Patient Perspective) to determine an overall disease control status.  ✔️ MM-aware dashboard and alerts: Automatically identifies patients with uncontrolled disease status through risk stratification. The system visualizes the combined burden of both conditions, allowing for a composite view that alerts when the multimorbidity requires medical attention.  ✔️ MM-aware digital recommendation: A Clinical Decision Support System (CDSS) utilizes cross-disease data patterns and daily symptom trends to assist physicians in individualizing therapy adjustments, particularly for severe cases where symptoms across domains interact. | ✔️ Pathophysiological interactions: Captures the biological link between upper and lower airways through simultaneous allergy screenings and pattern recognition.  ✔️ Treatment-related interactions: The CDSS provides guidance to harmonize therapy adjustments for both conditions simultaneously.  ✔️ Symptom and patient-reported interactions: Monitors the interaction of asthma and rhinitis symptoms (e.g., how nasal congestion correlates with bronchial control) to provide a unified assessment of disease control based on Patient-Reported Outcome Measures (PROMs).  ✔️Care process interactions: Integrates RAPP (quality-of-life assessments) into clinical workflows to address the total multimorbidity burden beyond isolated symptoms. |
| MATCH [31] | ✔️ ️ MM-aware analytics: Utilizes a digital cross-disease symptom diary to enable continuous monitoring of COPD and CHF statuses simultaneously.  ✔️ MM-aware dashboard and alerts: Supports timely interventions through integrated data tracking. The dashboard provides an integrated data view that allows for oversight of both conditions concurrently, enabling the identification of health status changes.  ✔️ MM-aware digital recommendation: A CDSS automatically generates personalized recommendations (e.g., relaxation exercises, calling a case manager) based on a standardized action plan. | ❌ Pathophysiological interactions: Not explicitly addressed via biological modeling. The system focuses on clinical symptoms and treatment rather than the underlying biological or physiological modeling of disease-to-disease links.  ✔️ Symptom and patient-reported interactions: Employs a cross-disease diary to track symptoms across multiple conditions for an integrated overview.  ✔️ Treatment-related interactions: Provides automated recommendations based on a standardized action plan that harmonizes care for both conditions.  ✔️ Care process interactions: Facilitates timely self-management and clinical interventions (e.g., calling a case manager) through standardized workflows. It bridges the gap between patient and clinician through structured communication protocols, such as automated prompts to contact a case manager when thresholds are met. |
| ATMoSPHÄRE [32] | ✔️ MM-aware analytics: Processes diverse geriatric assessments (functional and mental status) via a Decision Support System (DRS) to evaluate the overall condition of multi-morbid patients.  ✔️ MM-aware dashboard and alerts: Features a telemedical communication platform for continuous home-based monitoring and data integration.  ✔️ MM-aware digital recommendation: The DRS automatically generates tailored, need-based treatment recommendations based on specific patient targets and collected data. | ❌ Pathophysiological interactions: Not explicitly modeled at a biological level, but integrates multidimensional geriatric data to assess functional and mental decline.  ✔️ Symptom and patient-reported interactions: Captures interactions between various domains (e.g., how mental status impacts functional limitations) to monitor the overall condition profile.  ✔️ Treatment-related interactions: DRS provides individualized recommendations that harmonize treatment across multiple chronic conditions and geriatric syndromes.  ✔️ Care process interactions: Coordinates information flow between patients, informal carers, and professional staff through an integrated telemedical platform. |
| NOMHAD [33] | ✔️ MM-aware analytics: Aggregates Patient-Reported Outcome Measures (PROMs) and vital signs captured via mobile apps and connected devices. The system processes these data points to generate an Overall Risk Indicator (ORI), which reflects the patient’s integrated health status and accounts for interactions between coexisting diseases.  ✔️ MM-aware dashboard and alerts: Provides targeted visualizations on a web portal for physicians and call center nurses. This enables telemonitoring of health status changes and supports clinical decision-making by highlighting fluctuations in the ORI.  ❌ MM-aware digital recommendation: Not applicable. The system does not directly prompt specific automated actions or interventions. Instead, it provides the ORI as a decision-support tool to guide nurses in selecting appropriate manual actions, such as adapting care plans or advising emergency medical contact. | ✔️ Pathophysiological interactions: Accounts for biological interactions between coexisting diseases when calculating the overall risk status.  ✔️ Symptom and patient-reported interactions: Uses the ORI to aggregate indicators across multiple conditions into a single, comprehensive health profile.  ❌ Treatment-related interactions: Not explicitly addressed. The system focuses on risk assessment rather than providing automated therapy harmonization logic.  ✔️ Care process interactions: Acts as a triage tool to guide nurses in adapting care plans or advising emergency medical services based on the overall risk level. |
| mAFA [34] | ✔️ MM-aware analytics:Implements the ABC pathway to provide standardized, holistic assessments of AF-related multimorbidity. The system enables dynamic risk monitoring by regularly reassessing stroke and bleeding risks, alongside vital organ functions (e.g., kidney and liver function) that are critical for comorbid patients.  ✔️ MM-aware dashboard and alerts: Supports continuous monitoring of comorbidities like hypertension and AF symptoms through integrated digital tools. This provides clinicians with a real-time overview of the patient's stability and treatment targets.  ✔️ MM-aware digital recommendation: Provides structured clinical guidance for physicians based on guideline-based management and reassessed risk profiles. | ✔️ Pathophysiological interactions: The system dynamically monitors how AF and its treatments affect other organ systems (e.g., assessing how anticoagulation interacts with kidney and liver function).  ✔️ Symptom and patient-reported interactions: Integrates patient-reported symptoms and medical data to provide a holistic view of the AF-multimorbidity burden.  ✔️ Treatment-related interactions: Coordinates anticoagulation and comorbidity management (e.g., hypertension, diabetes) within a single, guideline-based care framework.  ✔️ Care process interactions: Uses the ABC pathway to structure an integrated care approach, facilitating proactive management and regular clinical reassessments. |
| mWellcare [35] | ✔️ MM-aware analytics: Processes cross-disease data collected by nurses during follow-up visits to evaluate five distinct chronic conditions simultaneously.  ✔️ MM-aware dashboard and alerts: While not characterized by real-time remote monitoring, it functions as a digital clinical support tool. It visualizes the collected data to provide a comprehensive overview during scheduled clinical encounters, ensuring no condition is overlooked.  ✔️ MM-aware digital recommendation: A Decision Support System (DSR) automatically generates integrated treatment plans for five conditions and lifestyle modification advisory based on standardized guidelines. | ❌ Pathophysiological interactions: Not explicitly modeled via biological interdependencies, but integrates clinical guidelines for five distinct conditions into a single management approach.  ✔️ Symptom and patient-reported interactions: Collects cross-disease data to provide a comprehensive overview of the patient's status across multiple chronic conditions.  ✔️ Treatment-related interactions: The DSR identifies and recommends harmonized treatment and lifestyle plans. This ensures that interventions (e.g., diet or exercise) are appropriate for the patient's entire multimorbidity profile rather than focusing on a single diagnosis.  ✔️ Care process interactions: Facilitates a structured workflow where nurses collect cross-condition data and physicians plan treatment steps based on integrated system prompts. |

References:

25. Deparis S, Pascale A, Tommasi P, Kotoulas S. An Analytical Method for Multimorbidity Management Using Bayesian Networks. Stud Health Technol Inform. 2018;247(247):820-824. [doi: 10.3233/978-1-61499-852-5-820] [Medline: 29678075]

26. Doyle J, Murphy E, Gavin S, et al. A Digital Platform to Support Self-management of Multiple Chronic Conditions (ProACT): Findings in Relation to Engagement During a One-Year Proof-of-Concept Trial. J Med Internet Res. Dec 15, 2021;23(12):e22672. [doi: 10.2196/22672] [Medline: 34914612]

27. Doyle J, McAleer P, van Leeuwen C, et al. The role of phone-based triage nurses in supporting older adults with multimorbidity to digitally self-manage - Findings from the ProACT proof-of-concept study. Digit Health. 2022;8(8):20552076221131140. [doi: 10.1177/20552076221131140] [Medline: 36238753]

28. Bousquet J, Schunemann HJ, Fonseca J, et al. MACVIA-ARIA Sentinel NetworK for allergic rhinitis (MASK-rhinitis): the new generation guideline implementation. Allergy. Nov 2015;70(11):1372-1392. [doi: 10.1111/all.12686]

29. Bousquet J, Devillier P, Anto JM, et al. Daily allergic multimorbidity in rhinitis using mobile technology: A novel concept of the MASK study. Allergy. Aug 2018;73(8):1622-1631. [doi: ] [Medline: 29569295]

30. Bousquet J, Arnavielhe S, Bedbrook A, et al. MASK 2017: ARIA digitally-enabled, integrated, person-centred care for rhinitis and asthma multimorbidity using real-world-evidence. Clin Transl Allergy. Dec 2018;8(1):45. [doi: 10.1186/s13601-018-0227-6]

31. Sloots J, Bakker M, van der Palen J, et al. Adherence to an eHealth Self-Management Intervention for Patients with Both COPD and Heart Failure: Results of a Pilot Study. Int J Chron Obstruct Pulmon Dis. 2021;16(16):2089-2103. [doi: 10.2147/COPD.S299598] [Medline: 34290502]

32. Borchers P, Piller S, Böhme M, Voigt K, Bergmann A. Need-based care of multi-morbid patients - supporting general practitioners with algorithm-generated recommendations of healthcare services (telemedicine-project ATMoSPHÄRE). BMC Fam Pract. Oct 8, 2021;22(1):198. [doi: 10.1186/s12875-021-01537-2] [Medline: 34625053]

33. Chacornac M, Faoro A, Texereau J, Billoet C, Hominal S. Performance of an eHealth (NOMHAD) System Comprising Telemonitoring, Telenotification, and Telecoaching for Patients With Multimorbidity: Proof-of-Concept Study. JMIR Form Res. Mar 11, 2022;6(3):e32205. [doi: 10.2196/32205] [Medline: 35275068]

34. Yao Y, Guo Y, Lip GYH, et al. The Effects of Implementing a Mobile Health–Technology Supported Pathway on Atrial Fibrillation–Related Adverse Events Among Patients With Multimorbidity. JAMA Netw Open. Dec 1, 2021;4(12):e2140071. [doi: 10.1001/jamanetworkopen.2021.40071]

35. Prabhakaran D, Jha D, Prieto-Merino D, et al. Effectiveness of an mHealth-Based Electronic Decision Support System for Integrated Management of Chronic Conditions in Primary Care: The mWellcare Cluster-Randomized Controlled Trial. Circulation. Jan 15, 2019;139(3):380-391. [doi: 10.1161/CIRCULATIONAHA.118.038192] [Medline: 30586732]
